# Live calcium imaging of *Aedes aegypti* neuronal tissues reveals differential importance of chemosensory systems for life-history-specific foraging strategies

**DOI:** 10.1186/s12868-019-0511-y

**Published:** 2019-06-17

**Authors:** Michelle Bui, Jennifer Shyong, Eleanor K. Lutz, Ting Yang, Ming Li, Kenneth Truong, Ryan Arvidson, Anna Buchman, Jeffrey A. Riffell, Omar S. Akbari

**Affiliations:** 1 Department of Entomology and Riverside Center for Disease Vector Research, Institute for Integrative Genome Biology, University of California, Riverside, Riverside, CA 92521 USA; 2 Department of Biology, University of Washington, Seattle, WA 98195 USA; 3 Section of Cell and Developmental Biology, University of California, San Diego, La Jolla, CA 92093 USA; 4 Tata Institute for Genetics and Society, University of California, San Diego, La Jolla, CA 92093 USA

**Keywords:** GCaMP6s, GECI, *Aedes aegypti*, Calcium, Neuronal, Stimuli-evoked responses

## Abstract

**Background:**

The mosquito *Aedes aegypti* has a wide variety of sensory pathways that have supported its success as a species as well as a highly competent vector of numerous debilitating infectious pathogens. Investigations into mosquito sensory systems and their effects on behavior are valuable resources for the advancement of mosquito control strategies. Numerous studies have elucidated key aspects of mosquito sensory systems, however there remains critical gaps within the field. In particular, compared to that of the adult form, there has been a lack of studies directed towards the immature life stages. Additionally, although numerous studies have pinpointed specific sensory receptors as well as responding motor outputs, there has been a lack of studies able to monitor both concurrently.

**Results:**

To begin filling aforementioned gaps, here we engineered *Ae. aegypti* to ubiquitously express a genetically encoded calcium indicator, GCaMP6s. Using this strain, combined with advanced microscopy, we simultaneously measured live stimulus-evoked calcium responses in both neuronal and muscle cells with a wide spatial range and resolution.

**Conclusions:**

By coupling in vivo live calcium imaging with behavioral assays we were able to gain functional insights into how stimulus-evoked neural and muscle activities are represented, modulated, and transformed in mosquito larvae enabling us to elucidate mosquito sensorimotor properties important for life-history-specific foraging strategies.

**Electronic supplementary material:**

The online version of this article (10.1186/s12868-019-0511-y) contains supplementary material, which is available to authorized users.

## Background

The yellow fever mosquito, *Aedes aegypti*, is a global vector of numerous debilitating arboviruses including Chikungunya, Dengue, Yellow Fever, and Zika [1]. Due to its ability to transmit copious pathogens, adaptability to diverse climates, flexibility in oviposition sites, and desiccation-tolerant eggs, *Ae. aegypti* are significant worldwide epidemiological burdens, leading to hundreds of millions of infections annually resulting in over 50,000 deaths [2–5]. To decrease the imposed global burden, many vector control methodologies have been developed and implemented, including a number of innovative genetic-based technologies such as the release of insects carrying dominant lethal (RIDL) [6] and the infection and introduction of mosquitoes harboring the intracellular bacterium, *Wolbachia* either spread into populations to reduce viral transmission [7, 8], or used for population suppression through *Wolbachia* induced cytoplasmic incompatibility (IIT) [9]. Moreover, there are also a number of innovative “gene drive” based technologies that are rapidly being developed in *Ae. aegypti* with the hope of making an impact in the future, in addition to innovative methods of generating sterile males using CRISPR [10–14]. Nonetheless, the most prevalent form of mosquito control used in the field today is the traditional use of chemical insecticides [15]. Although insecticides can have an impact on mosquito populations, due to their high costs, environmental impacts, requirements for continuous application, and rapid susceptibility to resistance [16], they are not sustainable long-term solutions. Therefore, significant efforts are necessary to discern the underlying molecular, genetic and physiological mechanisms important for arboviral vector competence with the overall aim of developing additional novel, insecticide-free methods to disrupt viral disease cycles [17].

At both larval and adult stages, mosquito sensory systems play pivotal roles in mediating a variety of behaviors, including locating food resources, habitat selection, and predator avoidance (Reviewed in [18–20]). As such, sensory systems provide attractive targets for suppressing vector behaviors at both the larval and adult stages. Over the years there have been numerous studies on adult mosquito sensory systems that have greatly advanced the field, such as the discovery of key olfactory and gustatory receptors [21, 22] as well as behavioral responses to host cues [23–25]. Notwithstanding, there remains critical gaps in understanding the direct relationships between sensorimotor and behavioral responses, specifically important for behaviors linked to vector competence such as host seeking and chemical avoidance. Additionally, only a handful of studies have focused on larval chemosensory systems resulting in significant gaps in a holistic understanding of mosquito sensory systems [26]. For example, olfaction is important for detecting long-range host cues in adult mosquitoes. However in an aquatic environment, either gustation, olfaction, or both, could detect long-range food indicators [27]. Food scarcity is an important ecological constraint on mosquito larvae [28], but little is known about the chemosensory mechanism of foraging in larval mosquitoes. Given the relative simplicity of the larval nervous systems, understanding chemosensory signal transduction, coding, and behavior in larvae could lead to novel control interventions and enable a more holistic understanding of mosquito behavior in areas such as food seeking and chemotaxis [19, 26].

Notwithstanding, as of recently, we have lacked effective genetic tools to study mosquito larval sensory systems as they process environmental information. Current tools used in mosquitoes to monitor neural activity include extracellular recording from sensilla and antennal lobe cells [29], as well as using synthetic calcium-sensitive dyes (e.g., FURA-2) in vivo, or in heterologous systems [30, 31]. To overcome the challenges of these existing approaches, here we have engineered *Ae. aegypti* to ubiquitously express a Genetically Encoded Calcium Indicator (GECI), termed GCaMP6s. GCaMP6s enables imaging of sensory-evoked calcium transients through changes in relative fluorescence [32]. Using this tool we gained the unprecedented ability to concurrently measure in vivo sensory responses and motor responses with high spatial and temporal resolution in regions of neuropil and muscles of live responding mosquitoes. This enabled us to generate functional insights into the importance of chemosensory channels in mediating behavior (e.g. foraging) by inactivating distinct olfactory and gustatory channels and measuring larval neural responses to diverse chemosensory stimuli in various genetic backgrounds such as those harboring mutations in important olfactory and gustatory receptors [21, 22]. Taken together, our results demonstrate the utility of GCaMP6s to link the sensory processing of specific stimuli to behavior responses of swimming larvae, thereby gaining a deeper functional understanding of mosquito multisensory integration.

## Results

### Development of an optogenetic-reporter of neuronal activity in *Ae. aegypti*

**Fig. 1:**
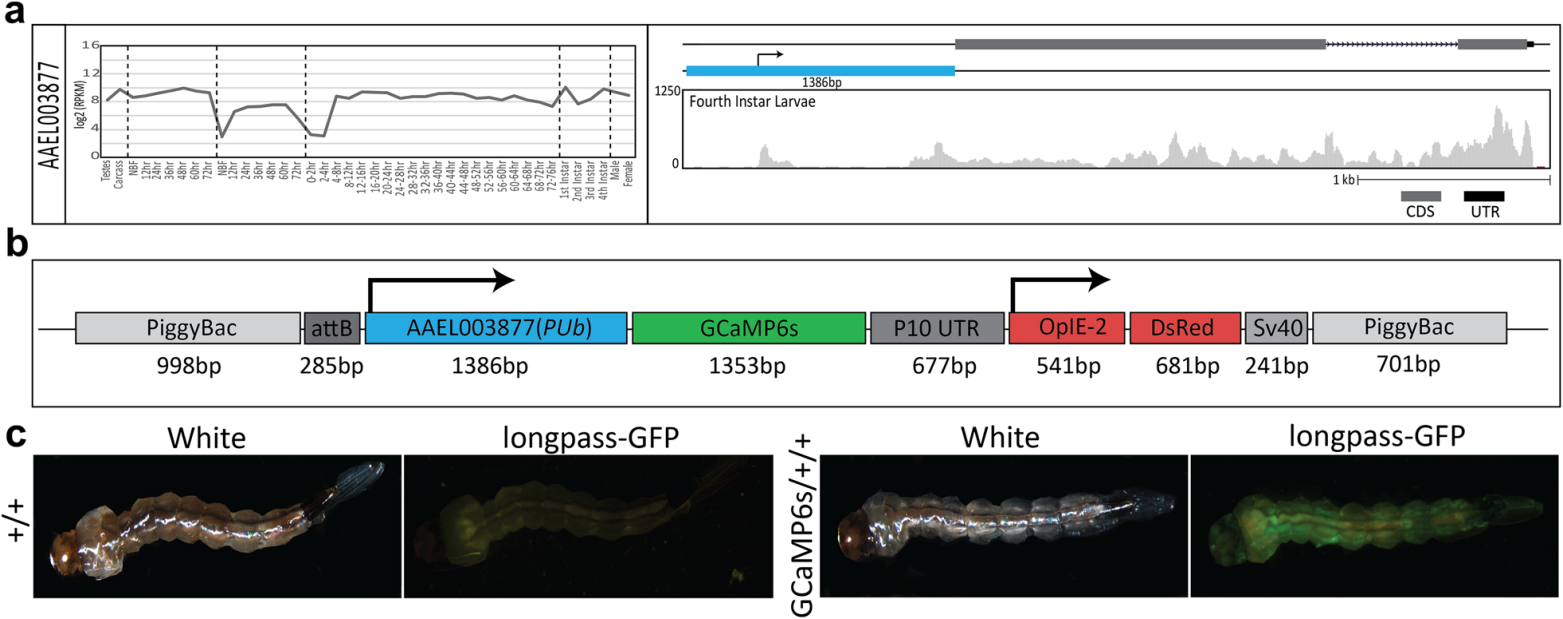
RNAseq expression, schematic representation of the GCaMP6s construct and larval fluorescence. Log_2_ (RPKM) expression values for the promoter, AAEL003877 (*PUb*) was plotted across development. Samples include, from left to right: testes; male carcasses (lacking testes); carcasses of females prior to blood feeding (NBF); female carcasses 12 h, 24 h, 36 h, 48 h, and 72 h post blood meal; ovaries from NBF females and at 12 h, 24 h, 36 h, 48 h, and 72 h post ecdysis; embryos from 0–2 h through 72–76 h; whole larvae from 1st instar, 2nd instar, 3rd instar and 4th instar; male pupae; and female pupae. A genome browser snapshot was with the Y axis showing expression level based on raw read counts of fourth instar larvae (**a**). A schematic representation of the *piggyBac*-mediated GCaMP6s construct. GCaMP6s is driven by AAEL003877(*PUb*)(blue) while dsRed by OpIE-2, the latter serving as a transgenic marker (**b**). Larval bright field images (*left*) and corresponding fluorescent images (*right*) show robust GFP transients throughout the whole body and DsRed fluorescence in the abdomen. +/+ represents wild-type larva. GCaMP6s/+/+ represents transgenic GCaMP6s larvae (**c**)

To visualize live calcium activity, we engineered a transgenic *Ae. aegypti* strain harboring genomic sources of a genetically-encoded calcium indicator, GCaMP6s [32]. To express GCaMP6s, we utilized the *polyubiquitin* promoter (AAEL003877, henceforth *PUb*), chosen for its generally high expression during nearly all developmental life stages and tissues as shown by previous promoter characterization experiments and developmental transcriptional profiling (Fig. 1a) [33, 34]. We inserted the *PUb* promoter upstream of the coding sequence for GCaMP6s within a randomly inserting *piggyBac* transposable element. Downstream to the *PUb* promoter driven GCaMP6s, we included an OpIE-2 promoter driving dsRed expression to serve as a robust transgenesis marker (Fig. 1b). To obtain a transgenic strain, the engineered *piggyBac* transgene was injected into the germ cells of 200 pre-blastoderm stage *Ae. aegypti* embryos (0–1 h old). Transgenic G1 mosquitoes harboring the transgene were readily identified by a bright expression of OpIE-2 driven dsRed in the abdomen, in addition to a robust calcium signaled activation of GCaMP6s in muscle and neural cells (Fig. 1c). To ensure that this strain represented a single chromosomal insertion, we backcrossed isolated individuals for four generations to wild-type (+/+) and measured Mendelian transmission ratios each generation and observed 50% of offspring inheriting the transgene, indicating that this strain likely represents a single chromosomal insertion. To precisely determine its genomic insertion location, we used inverse PCR and found the location of insertion to be on the 2nd chromosome with flanking 5′ and 3′ *piggyBac* regions positioned at AaegL5.0 reference (genomic loci 285,175,805–285,176,289 and 285,175,275–285,175,803, respectively. The location of the insertion site was mapped to a intronic region of an uncharacterized locus. To determine the fitness cost of our inserted transgene, we performed experiments comparing fertility, fecundity, egg hatching rate, and larval development time of our GCaMP6s inserted line to +/+. These experiments indicated that the transgene insertion did not significantly affect fertility (p = 0.4376), egg hatching rate (p = 0.1536), or larval development time (p = 0.2034), however fecundity was slightly increased (p < 0.05) (Additional file 1: Table S1).

### Temporal and spatial odor-evoked GCaMP6s responses in adults

To assess GCaMP6s functionality in a +/+ genetic background (termed GCaMP6s/+/+ from hereon), and to visualize sensorimotor activity elicited by specific sensory channels, we initially recorded and quantified calcium transients in adult mosquitoes that were stimulated with CO_2_. Distinct regions-of-interest (ROIs) were imaged across various sensory organs of adult mosquitoes using laser-scanning confocal microscopy. Calcium-evoked changes in fluorescence varied between sensory organs tested. For example, the tip of the maxillary palp displayed significant changes in fluorescence intensity across 4 replicates presumably related to the location of olfactory sensory neurons (OSNs) within capitate peg sensilla on the maxillary palp [22] (mean ΔF/F0 is 1.24 ± 0.13, *p* value = 0.0323, replicates 4) (Additional file 3: Figure S1A, Additional file 2: Video 1). While in the adult antennal flagellum, changes in fluorescence were recorded in the nodes between antennal segments (mean ∆F/F0 is 0.04 ± 0.31, p-value = 0.8240 and 0.01 ± 0.12, p-value = 0.9469, for ROI 1&2 respectively) and the internodes (meanΔF/F0 is − 0.08 ± 0.07, p-value = 0.1467), although neither were highly significant when comparing across 6 replicates (Additional file 3: Figure S1B, Additional file 4: Video 2). Additionally, when observing clusters of ommatidia within the adult eyes, changes in calcium signaled GCaMP6s activation were highly stochastic across 4 replicates presumably due to continuous optical responses as the mosquito is sensing the environment (mean ΔF/F0 is 0.02 ± 0.01, p-value = 0.4700; − 0.15 ± 0.20, p-value = 0.4398; 0.02 ± 0.04, p-value = 0.4949, for ROIs 1–3 respectively) (Additional file 3: Figure S1C, Additional file 5: Video 3). Although these results indicated that many regions (e.g. adult antennal flagellum and adult ommatidia) did not demonstrate significantly consistent odor-evoked responses across multiple replicates, accurate detection of subcuticular fluorescence was hindered by the adult’s thick cuticle and dense setation. To overcome this limitation in adults, we performed careful dissections of the head cuticle which enabled us to investigate the labeling efficacy of our GCaMP6s strain within the adult antennal lobe by examining several major cell types of potential interest to mosquito sensory processing. We found that GCaMP6s expression is sufficiently high to morphologically characterize diverse cell types including those within individual glomeruli, lateral cell cluster neurons, glia, and medial cell cluster neurons which will be invaluable for future studies (Additional file 6: Figure S2).

### Relative odor-evoked GCaMP6s responses in larvae

Compared to adults, 2nd instar larvae have relatively simplified neuroanatomical systems and a transparent cuticle making them well suited for detecting subcutaneous changes in fluorescence intensity reported by GCaMP6s without the need for dissections. These factors coupled with the limited knowledge regarding mosquito larval sensory responses motivated us to simultaneously image muscle and sensory calcium-evoked responses with the GCaMP6s/+/+ strain. Using either a 5 × or 10 × objective permitted us to record fluorescence in the whole body, or just the head capsule, respectively. Results from these experiments revealed significant changes in calcium transients within the longitudinal muscles within the 2nd abdominal segment across 4 replicates (meanΔF/F0 is 3.45 ± 0.57, p-value = 0.0011) (Additional file 3: Figure S1D, Additional file 7: Video 4) in the body in addition to the lateral retractors (mean ΔF/F0 is 2.04 ± 0.77, p-value = 0.004497), the deuterocerebrum (DE) across (mean ΔF/F0 is 0.45 ± 0.14, p-value = 0.002150) and medial retractors (mean ΔF/F0 is 2.57 ± 01.27, p-value = 0.013050) in the head (Additional file 3: Figure S1E, Additional file 8: Video 5). To further determine the cell type specificity of *PUb*-GCaMP6s expression within the larval brain, co-staining for GFP as well as either Glutamine Synthetase (GS) (which labels astrocyte-like glial) or alpha tubulin (which labels the nervous system) was performed [35]. Results exhibited colocalization between fixed GFP and both antibodies thus demonstrating that GCaMP6s under the *PUb* promoter expressed robustly in a variety of cell types (Additional file 9: Figure S3). Taken together, these results indicate that GCaMP6s can be used to effectively visualize sensorimotor activity in neural and muscle tissues of live mosquito larvae.

### Calcium imaging of odor-evoked responses in the larval brain in response to olfactory stimuli

**Fig. 2:**
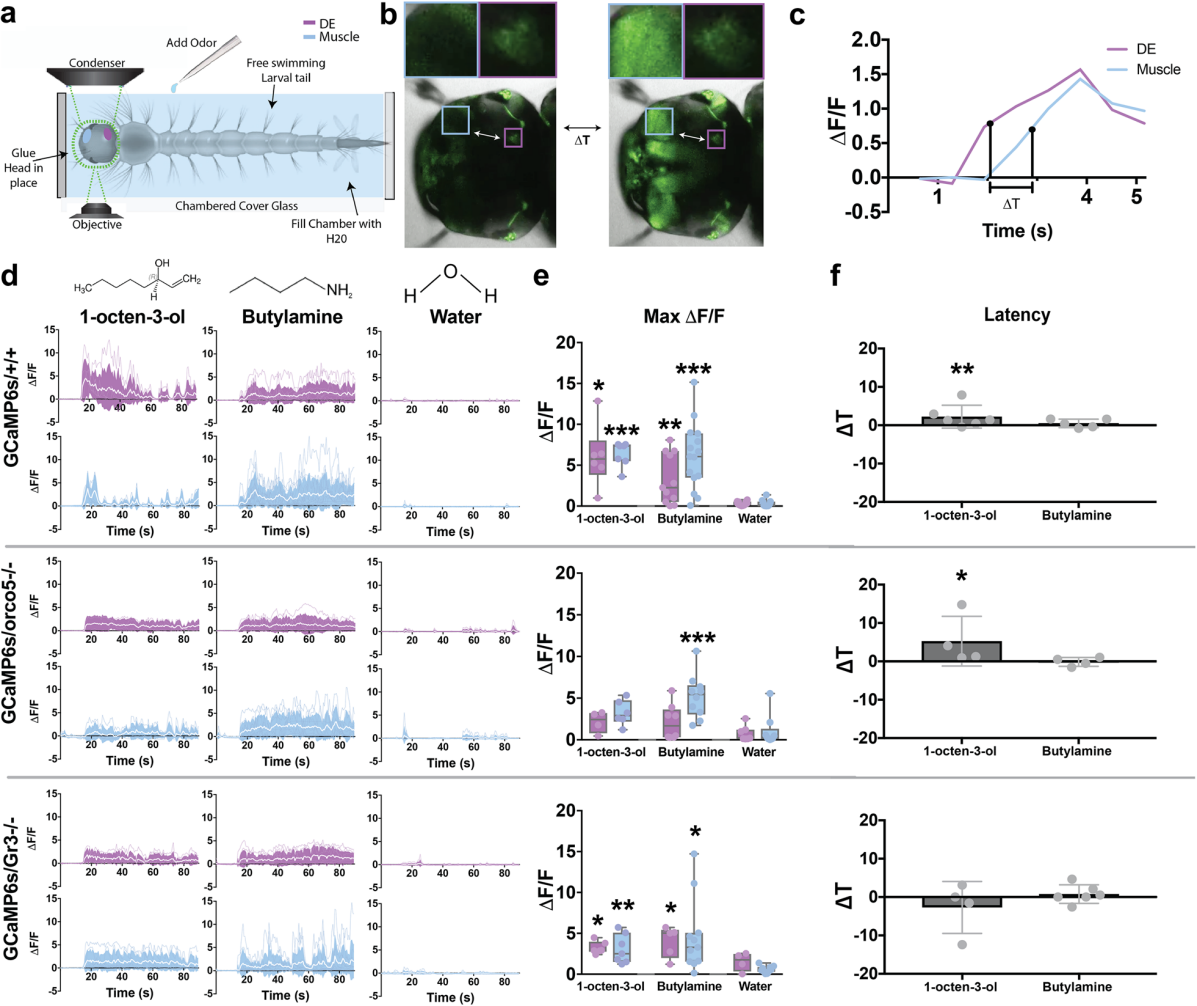
Live calcium imaging of stimulus-evoked responses in GCaMP6s/+/+, GCaMP6s/orco5−/−, and GCaMP6s/Gr3−/−. Mosquito larval calcium responses to various stimulants were recorded using a Leica SP5 Confocal microscope. To secure the larval head for imaging while allowing free movement of the larval tail, the dorsal side of the larva’s head was adhered to a chambered cover glass using quick setting adhesive. The chamber was then filled with water and the larvae was allowed to rest before being introduced to stimulants (**a**). To compare temporal difference between the deuterocerebrum (DE, purple) and muscles (blue), the difference between time points at 50% of the maximum ∆F/F of the first response peak following the addition of stimuli (**b**, **c**). Stimuli, including 1-octen-3-ol, butylamine, and water were introduced to GCaMP6s/+/+, GCaMP6s/orco5−/−, and GCaMP6s/Gr3−/− larvae 15 s after the start of recording. Calcium responses within the DE and muscles were recorded at 0.645 frames/sec (**d**) and maximum fluorescence values were plotted (**e**). The temporal difference in seconds between the DE and muscle responses were calculated and plotted by comparing DE and muscle timepoints at 50% of maximum ∆F/F (**f**). The number of biological replicates used for each experiment were 3 or greater. Differences in ∆F/F and Latency were analyzed using a Welch’s T-test and a Mann–Whitney U test respectively. *: p-value < 0.05, **: p-value < 0.01, ***: p-value < 0.001. (GCaMP6s/+/+ DE: 1-octen-3-ol n = 6; butylamine n = 11; water n = 12. GCaMP6s/+/+ Muscle: 1-octen-3-ol n = 7; butylamine n = 15; water n = 12. GCaMP6s/orco5−/− DE: 1-octen-3-ol n = 4; butylamine n = 8; water n = 7. GCaMP6s/orco5−/− Muscle: 1-octen-3-ol n = 6; butylamine n = 10; water n = 9. GCaMP6s/Gr3−/−: 1-octen-3-ol n = 6; butylamine n = 11; water n = 12. GCaMP6s/Gr3−/− DE: 1-octen-3-ol n = 6; butylamine n = 5; water n = 4, GCaMP6s/Gr3−/− Muscle: 1-octen-3-ol n = 7; butylamine n = 11; water n = 10. Latency of GCaMP6s +/+ response: 1-octen-3-ol n = 6; butylamine n = 5. Latency of GCaMP6s/orco5−/− response: 1-octen-3-ol n = 4; butylamine n = 4. Latency of GCaMP6s/Gr3−/− response: 1-octen-3-ol n = 4; butylamine n = 6.)

**Fig. 3:**
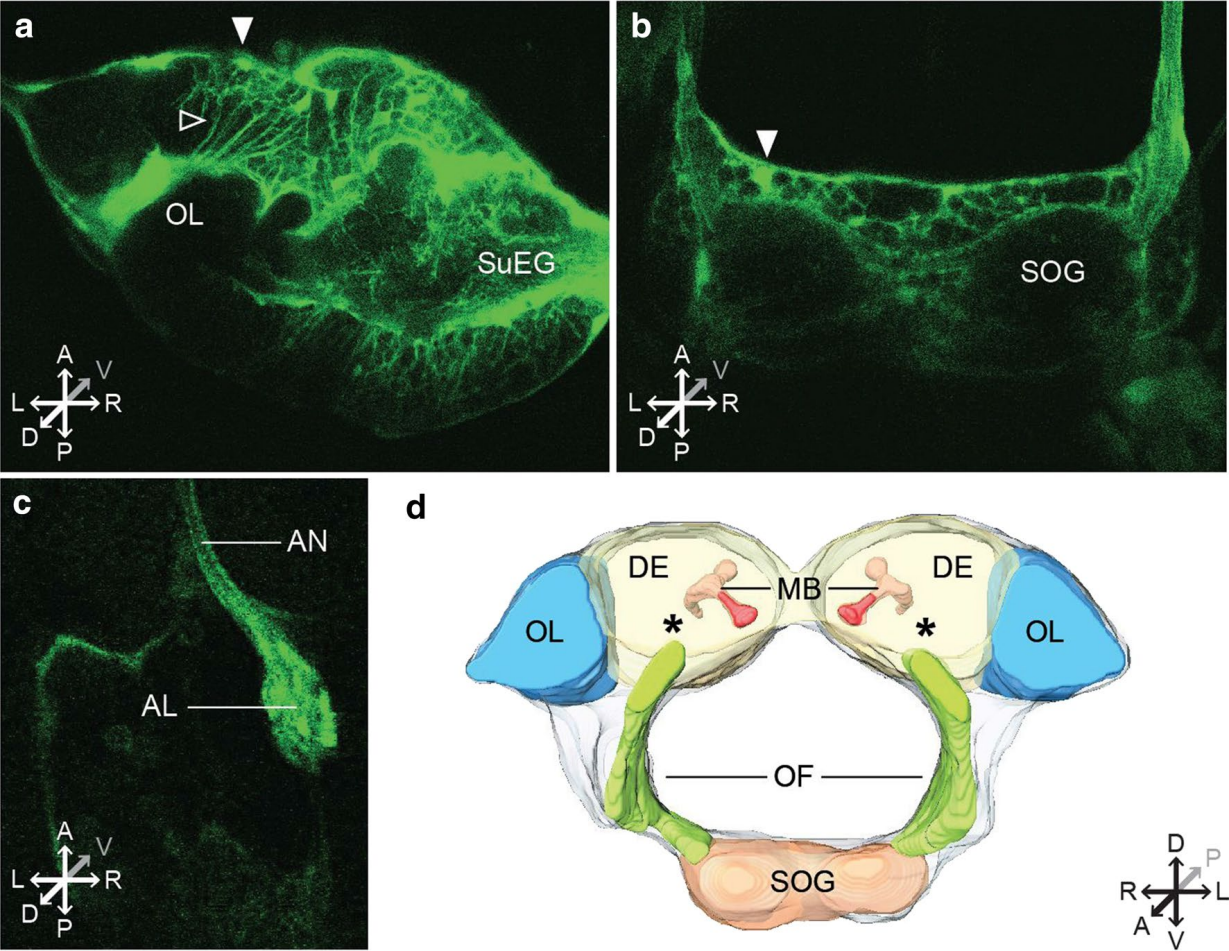
Two-photon imaging of *Ae. aegypti* larvae. Example images of two-photon microscopy imaging of live *Ae. aegypti* larvae. Areas of interest included the optic lobe; OL, supra-esophageal ganglion; SuEG (upper DE, (**a**)), subesophageal ganglion; SOG (**b**), antennal nerve; AN and antennal lobe; AL. Cell bodies (labeled with a filled arrow), neuropil (labeled with an open arrow). (**c**). [A; B: L4 larvae with dorsal head cuticle removed. **c** L2 larva imaged through transparent cuticle]. **d** Approximate 3D reconstruction of larval brain regions based on a confocal scan of the dissected larval brain. Asterisks indicate the rough location of the antennal lobe. Additional labeled brain regions include a general schematic of the optic lobe (OL), deutocerebrum (DE), mushroom bodies (MB), suboesophageal ganglion (SOG), and oesophageous foramen (OF) [35], Thermo Scientific Amira Software)

To gain a more comprehensive understanding of the links between stimulus-evoked calcium responses in the brain and muscles of the larval head, a novel, minimally invasive, tethered-swimming assay was developed. This assay consisted of adhering the dorsal side of the larval head to a chambered cover glass, thereby immobilizing the head, while the larva was submerged in enough water to enable constant imaging of calcium transients within the head capsule while the tail could freely swim and the breathing tube could siphon oxygen (Fig. 2a). Importantly, the larval head capsule is strikingly transparent requiring no surgical removal of cuticle thus enabling the larva to survive for extended periods (up to 48 h) permitting multiple recordings on the same individual. Responses in the DE and lateral abductors were analyzed as representatives of neural and muscle responses, respectively with at minimum 3 biological replicates per stimuli. Stimuli tested included chemicals previously shown to be relevant to adult mosquitoes such as a known olfactory receptor agonist (2-(4-Ethyl-5-(pyridin-3-yl)-4*H*-1,2,4-triazol-3-ylthio)-*N*-(4-ethylphenyl) acetamide, henceforth VUAA1) [36], attractants (1-octen-3-ol, ethyl acetate and lactic acid) [37–40], a known exciter of multiple groove-peg OSNs (butylamine) [27], and other behaviorally relevant compounds (sucrose, lobeline, glutamate, fish food) [41, 42]. All stimulants were prepared at 6 × 10^−5^M, with the exception of fish food (see “Selection and preparation of odorants”). Previous studies have demonstrated mosquito larval response to various stimuli at concentrations ranging from 10^−5^ to 10^−2^M [26], here we used the bottom range of concentrations in order to prevent overstimulation within our small chamber. By stimulating GCaMP6s/+/+ larvae to this panel of chemicals we found that there were significant calcium responses in the DE to several stimuli including 1-octen-3-ol (max ΔF/F0 is 6.09 ± 3.85, p-value = 0.0145), butylamine (max ΔF/F0 is 3.29 ± 3.05, p-value = 0.0092), ethyl acetate (max ΔF/F0 is 3.14 ± 2.61, p-value = 0.0077), lobeline (max ΔF/F0 is 2.57 ± 2.17, p-value = 0.0224), lactic acid (max ΔF/F0 is 2.31 ± 1.72, p-value = 0.0366), and VUAA1 (max ΔF/F0 is 2.12 ± 1.66, p-value = 0.0458), while sucrose (max ΔF/F0 is 2.01 ± 2.70, p-value = 0.1053), glutamate (max ΔF/F0 is 0.79 ± 0.61, p-value = 0.1632), and fish food extract (max ΔF/F0 is 0.62 ± 1.02, p-value = 0.6101) did not display significant changes in fluorescence intensity when compared to responses evoked by water (Fig. 2d, e, Additional file 10: Figure S4, Additional file 11: Figure S5A). Interestingly, 1-octen-3-ol, a known mosquito adult attractant produced by microbes [43], displayed the greatest calcium response, followed by butylamine and ethyl acetate, with the former previously documented to induce a response in activated grooved-peg OSNs in *Anopheles gambiae*, *Anopheles quadriannulatus*, and *Culex quinquefasciatus* [27, 44]. Moreover, when observing muscle responses to the same stimuli we observed significant calcium increases in 5 of the 6 stimuli that also displayed significant responses in the DE. These stimuli included 1-octen-3-ol (max ΔF/F0 is 6.38 ± 1.50, p-value = 2.91e-05), butylamine (max ΔF/F0 is 6.11 ± 4.04, p-value = 4.11e-05), ethyl acetate (max ΔF/F0 is 3.10 ± 2.88, p-value = 0.00096), lobeline (max ΔF/F0 is 2.41 ± 2.45, p-value = 0.00408), and VUAA1 (max ΔF/F0 is 3.16 ± 2.16, p-value = 0.00437). Similar to the DE, the muscles did not exhibit significant responses to sucrose (max ΔF/F0 is 1.04 ± 1.64, p-value = 0.07634), glutamate (max ΔF/F0 is 0.68 ± 1.07, p-value = 0.1453), or fish food (max ΔF/F0 is 0.59 ± 0.45, p-value = 0.1534) when compared to responses evoked by water. Contrary to results from the DE, responses by muscles to lactic acid (max ΔF/F0 is 1.53 ± 1.88, p-value = 0.07509) were not significant. Lastly, the universal expression of GCaMP provided an opportunity for temporal comparison between brain and muscle responses. When analyzing the latency in response between the DE and muscles (Fig. 2b, c), 1-octen-3-ol elicited a significant latency of 2.24 ± 3.19s (p-value = 0.003036), while butylamine elicited a latency of 0.48 ± 1.11s (p-value = 0.2867) (Fig. 2f). Furthermore, a persistence in response to 1-octen-3-ol was seen in the DE but not in the muscle. This contrasted with the response to a majority of the other stimuli including butylamine where fluorescent expression in the muscle matched that of the DE (Additional file 12: Figure S8). To further explore the potential functionality of our GCaMP6s mosquito line, we used two-photon microscopy to investigate higher spatial resolution in imaging GCaMP6s expression. Although this experimental protocol was unsuitable for imaging stimulus-evoked responses due to the substantial movement of the brain, we were able to image various regions of interest throughout the brain of live, head-fixed larvae (Fig. 3), demonstrating that this technique may be useful for future studies in larval neurobiology such as larval vision or nociception.

### Larval brain responses to olfactory stimuli in mutant genetic backgrounds

To gain further insight into the genetic basis for neuronal responses to various stimuli, we genetically introgressed GCaMP6s/+/+ mosquitoes into two separate genetic backgrounds that harbored homozygous viable mutations in either an important odorant coreceptor required for odor detection (orco; [21]) or a subunit of the heteromeric CO_2_ receptor (Gr3−/−; [22]) (Additional file 13: Figure S6). Previous studies have demonstrated that orco is a highly conserved subunit of ORs that influences multiple odorant receptors and plays a role in the discrimination between different host organisms’ olfactory cues. Gr3 on the other hand has been noted to play a major role in CO_2_ detection, thus also affecting host detection. Using our larval tethered-swimming confocal imaging assay and stimulus panel described above to compare calcium evoked responses between GCaMP6s/+/+, GCaMP6s/orco5−/−, GCaMP6s/Gr3−/− enabled us to parse out receptors important for eliciting responses to various stimuli. Interestingly, we found that, compared to GCaMP6s/+/+, the DE and muscles of GCaMP6s/orco5−/− elicited fewer significant responses to stimuli (p-value > 0.05) as well as a general reduction in calcium evoked responses to all stimuli (Additional file 6: Figure S2, Additional file 9: Figure S3B). Only butylamine elicited significant responses compared to the water control in the muscles (max ΔF/F0 is 5.20 ± 2.60, p-value = 8.55e-4) (Fig. 2e, Additional file 11: Figure S5B). Additionally, when comparing DE and muscle responses between GCaMP6s/orco5−/− and GCaMP6s +/+, only muscle responses to 1-octen-3-ol and VUAA1 within GCaMP6s/orco5−/− showed a significant decrease (Additional file 11: Figure S5E). A strong decrease in DE response to 1-octen-3-ol was also seen in GCaMP6s/orco5−/−, however it was not significant (max ∆F/F is 2.14 ± 1.30, p-value = 0.0563). Examining the latency of response in GCaMP6s/orco5−/− demonstrated that stimulation with 1-octen-3-ol elicited responses in muscles 5.27 ± 6.50 s after activation of the DE (p-value = 0.04412), while butylamine showed little difference in the latency between DE and muscle response (0.13 ± 1.14 s; p-value = 0.9492) (Fig. 2f). Furthermore, although the response intensity to 1-octen-3-ol in the DE was not as strong as that of GCaMP6s +/+, the response was seen to persist for a longer period of time (Additional file 12: Figure S8). Taken together, our results demonstrate orco’s role in the detection of numerous chemosensory stimuli. Additionally, we found that orco may play an important role in 1-octen-3-ol detection and response. A nonsignificant reduction of response in the DE yet a significant reduction in the muscles indicate that even strong yet insignificant decreases in neural response may lead to a significant reduction of muscle output.

In contrast to the GCaMP6s/orco5−/− mutants, GCaMP6s/Gr3−/− mutants showed more robust calcium-evoked responses, and were generally not significantly different from those of GCaMP6s/+/+ with the exclusion of muscle responses to 1-octen-3-ol (Additional file 11: Figure S5C–E). For instance, relative to the water control, 1-octen-3-ol and ethyl acetate elicited strong responses in both the DE and muscle (p-value < 0.05) (Additional file 10: Figure S4). In total, five stimuli evoked significant increases in fluorescence within muscles of GCaMP6s/Gr3/−/− mutants; including 1-octen-3-ol (max ΔF/F0 is 3.08 ± 1.79, p-value = 0.009765), butylamine (max ΔF/F0 is 4.59 ± 4.46, p-value = 0.01383), ethyl acetate (max ΔF/F0 is 4.51 ± 1.85, p-value = 0.001199), VUAA1 (max ΔF/F0 is 1.86 ± 1.11, p-value = 0.01393), and sucrose (max ΔF/F0 is 2.03 ± 1.47, p-value = 0.04154)(Additional file 11: Figure S5C). Interestingly, the latency in response between the DE and muscle ROIs were near-simultaneous for butylamine (0.78 ± 2.61s, p-value = 0.6565) and 1-octen-3-ol (2.72 ± 6.75s, p-value = 0.9389), with the latter also demonstrating more persistent responses in both the DE and muscles, suggesting that gustation or other chemosensory channels may be involved in the processing of these odorants (Fig. 2f, Additional file 12: Figure S8).

### Odor-evoked behavior in free-swimming larvae

**Fig. 4:**
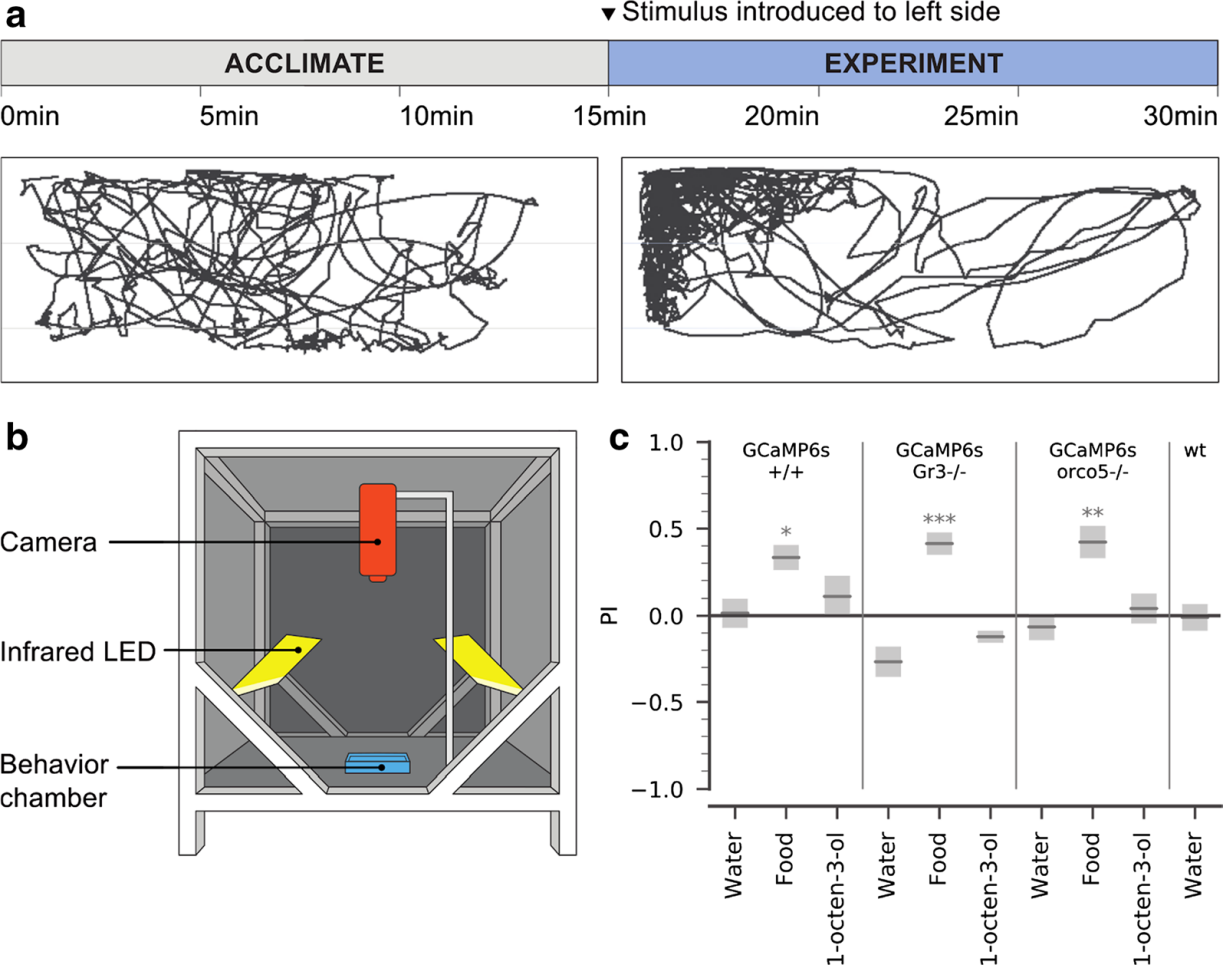
Behavioral analysis of stimulus-evoked responses in GCaMP6s/+/+, GCaMP6s/orco5−/−, GCaMP6s/Gr3−/−, and wt larvae. **a** In each experiment, the larva was allowed to acclimate in the arena for 15 min. Next, 100 µL of one stimulus was introduced to the upper left side of the arena. In both stages, larval behavior was recorded at 2fps, and larval position in each frame was extracted using ImageJ and Python. This example trajectory shows the movement of a GCaMP6s/orco5−/− larva before and after the addition of 100 µL food extract. **b** The dark experimental arena used for behavior testing. Animals were released individually into a custom 3D printed porcelain behavior chamber (blue), lit with infrared LED panels (yellow) and recorded with a Basler Scout Machine Vision Area Scan GigE camera (orange). **c** Using these trajectories, we compared PI (defined as the proportion of time spent in the odor half of the arena minus the proportion of time spent in the non-odor half) across all larvae during the acclimation and experiment phase. Gray bars show mean ± SEM during the experiment phase. p-values: pairwise T-test comparing acclimation period to experiment period. *: p-value < 0.05, **: p-value < 0.01, ***: p-value < 0.001. GCaMP6s/+/+: water n = 20; 1-octen-3-ol n = 14; food extract n = 20. GCaMP6s/orco5−/−: water n = 24; 1-octen-3-ol n = 16; food extract n = 20. GCaMP6s/Gr3−/−: water n = 16; 1-octen-3-ol n = 17; food extract n = 16; Liverpool wt: water n = 19

Previous studies have shown that mosquito larvae respond behaviorally to chemosensory stimuli including 1-octen-3-ol [26], but the genetic basis of these responses remain unclear. To investigate the behavioral responses of the GCaMP6s larvae in various genetic backgrounds (GCaMP6s/+/+, GCaMP6s/ocro5−/−, and GCaMP6s/Gr3−/−), we examined free-swimming larval responses to a limited odor panel in a custom arena. Individual larvae were allowed to acclimate inside the dark behavior arena before a stimulus - either food extract, 1-octen-3-ol, or a water-only (negative) control - was added to one side of the arena, and responses were analyzed and compared for the 15-minute acclimation period and the following 15-minute experiment period (Fig. 4). From the videos, we were able to quantify each larva’s preference index (PI, defined as the proportion of time spent in the odor half of the arena minus the proportion of time spent in the non-odor half) (Fig. 4). Importantly, prior to stimulation we found no differences in mean speed between larvae of the mutant backgrounds (Additional file 14: Figure S7), suggesting that these mutant backgrounds are not impaired in motility. In all strains, the addition of water had no significant influence on which side of the chamber the larvae preferred (p > 0.05, pairwise t-test compared to acclimation period). Larvae of all strains significantly preferred the side of the chamber with the food extract (p < 0.05), and this preference was not significantly different across strains (p > 0.05, 2-way ANOVA by strain and odor). Larvae of all three strains showed no significant positional preference for 1-octen-3-ol (p > 0.05).

## Discussion

In these experiments, we have expanded the toolbox of techniques for investigating a globally important disease vector, *Ae. aegypti*, and explored the potential applications of these tools for investigating overarching questions in neurobiology such as sensory integration and information processing. Furthermore, our results from behavioral experiments suggest interesting avenues of future research in *Ae. aegypti* chemosensory processing. The robust expression of GCaMP6s in various mosquito tissues (Fig. 1) allows quantification of stimulus-evoked responses in both motor and sensory systems, and in the adult and larval stages, including the adult antennae, adult maxillary palps, larval deutocerebrum (DE), and larval muscle (Fig. 2, Additional file 3: Figure S1). This broad GCaMP6s expression allowed us to investigate both motor and sensory responses in *Ae. aegypti* larvae to an ecologically relevant panel of chemosensory stimuli. These cues elicited spatiotemporal patterns in GCaMP6/+/+ larval muscle and central nervous system (CNS), and revealed that key components of natural odors may be relevant across *Ae. aegypti* life stages (Additional file 3: Figure S1). In addition, monitoring both muscle and neural response allowed for visualization of stimuli specific relationships between the sensory and motor responses. Some stimuli elicited a neural response followed by a muscle output, however some demonstrated a neural response with no muscle output possibly due to a lack of behavior related to the stimulus. Also some stimuli generated a muscle response without the detection of a neural response possibly due to neural processing of that stimulus within a different region of the brain outside of our imaging plane. Further, when we crossed this GCaMP6s/+/+ line with orco5−/− mutant to generate GCaMP6s/orco/−/−, we observed attenuation in these stimulus-evoked responses, particularly in response to known OR ligands (VUAA1 [45], 1-octen-3-ol [46], Additional file 10: Figure S4). By contrast, GCaMP6s/Gr3−/− larvae showed no significant impairment in response to any of the odorants tested, indicating that the heteromeric CO_2_ receptor complex is not critical for the detection and response to these stimuli in the larval stage. This supports previous transcriptome work suggesting that the Gr3 receptor is expressed at very low levels in *Ae. aegypti* larvae [47]. Together, these results demonstrate the utility of these GCaMP6s/mutants for investigating the neural representation of chemosensory-mediated stimuli. Interestingly, our neuronal imaging showed no significant response to food odors, however muscle responses were observed. This may reflect the fact that our dorsal imaging plane did not extend into the ventral sub-oesophageal ganglion (SOG), which is innervated by sensory nerves from the mouthparts. Future experiments may look into imaging additional neuropils to detect any possible neural responses. Finally, our behavioral experiments contextualized some of these stimulus-evoked responses in a more naturalistic environment, revealing that ORs may act in parallel with other chemosensory channels during foraging behavior in *Ae. aegypti* larvae (Fig. 4). Together, our combination of calcium imaging and behavior experiments highlights the importance of studying chemosensory behavior from multiple perspectives, and build on earlier work on the chemosensory repertoire [26, 48] and behaviors of mosquito larvae [40, 49] to gain a more complete understanding of mosquito chemical ecology.

Although our GCaMP6s +/+ line has demonstrated the ability to generate useful insight in mosquito chemosensation, there remains a number of limitations that should be addressed if used for future research. Firstly, due to the nature of the *PUb* promoter, GCaMP6s expression can occur in all types of cells. The lack of specificity may make interpreting results and expression patterns difficult. For example, we observed muscle responses to VUAA1 (an olfactory receptor agonist) which likely represent activation of other sensory channels and subsequent downstream responses in the motor system rather than direct stimulus-evoked olfactory responses. Nevertheless, for the purpose of our experiments, the broad expression of GCaMP6s allowed for a general and holistic overview of a chemosensory responses to various stimuli. For future experiments that may want to utilize this strain to examine responses from specific cell types or neurons, it can be complemented with other neuronal recording techniques, such as patch clamp, to allow for the accurate quantification of specific cells while also the ability to generally visualize responses surrounding the cell of interest.

## Conclusions

Our results highlight important avenues of future research in mosquito sensory processing. First, the mechanisms of chemosensory cue detection in *Ae. aegypti* larvae remains an open question. In terrestrial environments, long-range chemosensory stimuli are largely limited to volatile compounds with high vapor pressure at ambient temperatures [50]. However, *Ae. aegypti* larvae inhabit an aquatic environment that is a rich source of chemical signals far more varied in size, polarity, and structure, such as large proteins, amino acids, long hydrocarbon chains, and multi-molecular fragments of organic debris [51]. Interestingly, *Ae. aegypti* larvae express far fewer ORs than adults [48] and have a markedly smaller and physiologically less developed antennal lobes [35, 52]. *Ae.aegypti* larvae may rely on a more diverse assortment of IRs and GRs, in addition to ORs, to detect a wide range of water-borne chemicals relevant to behaviors such as foraging and predator avoidance [28, 53]. Characterization of the *Ae. aegypti* larval IRs and GRs may help identify chemical compounds that are most relevant to larval environments, and lend insight into the spectrum of larval chemical receptors. In addition to receptor-level chemical detection, the mechanism of chemosensory processing in the *Ae. aegypti* larval CNS is not well understood. Aquatic crustaceans integrate information from hydrodynamic detectors and two distinct types of chemosensory receptors within the CNS [54, 55], but it is unclear if *Ae. aegypti* sensory transduction follows this same model. From an evolutionary perspective, comparing the mechanism of *Ae. aegypti* larval olfaction to crustacean, amphibian, and fish models may also provide critical insight into the convergent evolution of aquatic chemosensation. Our *Ae. aegypti* GCaMP6/+/+ strain is of particular interest as it is, to our knowledge, the first example of GCaMP6 expression in an aquatic insect model.

Additionally, some odor components are shared among multiple ecologically relevant cues for mosquitoes, and neurobiological implications of these correlations are unclear. For example, 1-octen-3-ol is a component of both host odors [56] and microbial byproducts [57] that may function as food for larval mosquitoes. It is not unreasonable to hypothesize that there may be strong evolutionary selection on mosquito ORs that are beneficial in both life-history stages, and if so, identifying those chemicals that operate as both larval attractants and adult host cues may provide attractants that can be leveraged for mosquito control. Moreover, the mechanism of chemotaxis in *Ae. aegypti* larvae remains an open question. In other insect models such as *D. melanogaster*, larvae employ active sampling strategies to locate and navigate to food cues [58]. But it is unclear how *Ae. aegypti* larvae navigate chemosensory signals in an aquatic environment that is quite different in volume and turbidity from those experienced by *D. melanogaster,* or even *E. coli [*59*]* and *C. elegans [*60*]*, which navigate chemosensory gradients at a significantly smaller scale. Quantitative modeling and further behavioral experiments may help better understand chemotaxis in an enigmatic aquatic insect model, and highlight interesting commonalities and differences in navigation strategy across different environments and spatial scales.

Generalizing further, the GCaMP6s/Gr3−/− and GCaMP6s/orco5−/− mutants could address critical gaps in our broader understanding of multisensory integration and sensorimotor responses, particularly in adult mosquitoes. Behavioral work in *Ae. aegypti* adults presents compelling evidence for the involvement of multisensory integration in host-seeking [22, 23]. However, little is known about the neural bases of these behaviors. In *D. melanogaster*, GCaMP6s imaging has revealed the functional basis of information convergence in higher-order brain areas [61, 62]. Future work with GCaMP6s/+/+ may similarly help decode the neural representations of multimodal host cues in mosquitoes, and provides motivation for the development of transcriptional control systems such as GAL4/UAS or the Q-system [63] for tissue-specific GCaMP6s expression. Importantly, we observed high GCaMP6s expression in both muscle and neuropil (Fig. 2, Additional file 12: Figure S8). In *D. melanogaster*, concurrent analysis of neural response and motor output has facilitated experiments in the integration of sensory processing and sensory-motor transformations [64–69]. By taking advantage of this simultaneous recording capacity in *Ae. aegypti*, additional experiments could investigate how these multisensory integration pathways mediate motor responses, and ultimately, determine behavioral decisions such as host choice and oviposition site preference. Finally, these GCaMP6s/+/+ mosquitoes and GCaMP6s/Gr3−/− and GCaMP6s/orco5−/− mutants could provide additional information and strategies for the control of disease-vector mosquitoes. Female mosquitoes may use olfactory indicators of larval habitat quality to choose oviposition sites [70]. A better understanding of chemosensory cues that elicit strong responses in larvae could help identify new attractants for use in oviposition traps, or oviposition deterrents for use in homes and outdoor water containers.

## Materials and methods

### Insect rearing

Mosquitoes used in all experiments were derived from of the *Ae. aegypti* Liverpool strain, which was the source strain for the reference genome sequence. Mosquitoes were raised in incubators at 28 °C with 70–80% relative humidity and a 12 h light/dark cycle. Larvae were fed ground fish food (TetraMin Tropical Flakes, Tetra Werke, Melle, Germany) and adults were fed with 0.3 M aqueous sucrose. Adult females were blood fed three to five days after eclosion using anesthetized mice. All animals were handled in accordance with the guide for the care and use of laboratory animals as recommended by the National Institutes of Health and supervised by the local Institutional Animal Care and Use Committee (IACUC).

### Construct assembly

To generate the GCaMP6s plasmid (plasmid sequence and DNA available for order at addgene ID# 106868), components were cloned into the *piggyBac* plasmid pBac-3xP3-dsRed [71] using Gibson assembly/EA cloning [72]. Specifically, pBac-3xP3-dsRed was digested with BstBI and SacII, and an attB site, amplified from a stock attB plasmid [73] with primers 997.C5 and 997.C6. The predicted *Ae. aegypti polyubiquitin* (*PUb*) promoter fragment [33] was amplified from *Ae. aegypti* genomic DNA using primers 997.C1 and 997.C2. While the GCaMP6s fragment [32] was amplified from vector pGP-CMV-GCaMP6s (Addgene plasmid #40753) using primers 997.C3 and 997.C4 and cloned in via EA cloning. The resulting plasmid was then digested with PacI and AvrII and the following fragments cloned in via EA cloning. P10 3′UTR [74] was amplified with primers 997.C7 and 997.C8 from vector pJFRC81-10XUAS-IVS-Syn21-GFP-p10 (Addgene plasmid 36432) and the OpIE-2 promoter region [75] amplified from vector pIZ/V5-His/CAT (Invitrogen) using primers 997.C9 and 997.C10. The plasmid was grown in strain JM109 chemically competent cells (Zymo Research #T3005) and isolated using Zyppy Plasmid Miniprep (Zymo Research #D4037) and maxiprep (Zymo Research #D4028) kits. The full plasmid sequence was verified using Source Bioscience Sanger sequencing services. A list of primer sequences used in the above construct assembly can be found in Additional file 15: Table S2.

### Generation of GCaMP6s/+/+, GCaMP6s/Gr3−/−, and GCaMP6s/orco5−/− transgenic lines

GCaMP6s/+/+ mosquitoes were created by injecting 200 0–1 h pre-blastoderm stage embryos with a mixture of the GCaMP6s plasmid described above (200 ng/µl) and a source of *piggyBac* transposase (phsp-Pbac, (200 ng/ul)) [76–79]. Embryonic collection and microinjections were largely performed following previously established procedures [71, 80]. Injected embryos were hatched in deoxygenated water and surviving adults were placed into cages. Adult G0 females were allowed a blood-meal 4 days after eclosion. Following general rearing procedures described above, 3000 G1 larvae were screened for expected fluorescent markers, OpIE-2-dsRed, and *PUb*-GCaMP6s (Fig. 1c, Additional file 13: Figure S6D). Larvae with positive fluorescent signals were collected under a fluorescent stereomicroscope (Leica M165FC). All positive larvae collected produced consistent *PUb*-GCaMP6s and OpIE-2-dsRed expression patterns hinting that these larvae contained the same insertion. Only one transgenic line was found. To strengthen our belief that this line was produced with a single chromosomal insertion, single individuals from each of the lines were backcrossed for four generations to our wild-type stock. Mendelian transmission ratios for each generation were measured. In all cases, we observed a 50% transmission ratio in each generation, indicating that our strain likely represented an insertion on a single chromosome. To obtain a nearly complete homozygous line, our GCaMP6s line was screened and selected for at least 20 generations. For each generation, wild-type individuals were removed and the remaining GCaMP6s +/+ individuals were mated together until the offspring from a colony reached nearly 99% GCaMP6s +/+ when screened. To obtain the GCaMP6s/orco5−/− homozygous line, GCaMP6s/+/+ (♂) was crossed with orco5−/− (♀), then G1 individuals (♂) with the GCaMP6s phenotype were backcrossed with orco5−/− individuals (♀) for at least 8 generations as single mosquito pairwise matings, sanger sequencing was utilized to confirm GCaMP6s/orco5−/− homozygosity (Additional file 13: Figure S6A, B). Following, single pair matings between GCaMP6s/orco5−/− individuals were conducted and screened for 100% inheritance of transgenic markers thus creating a line fully homozygous for orco5−/− and nearly homozygous for GCaMP6s. To obtain the GCaMP6s/Gr3−/− mutant homozygous line, GCaMP6s/+/+ (♂) was crossed with GR3−/− (♀, labeled with a CFP marker), then continually selected for individuals with correct markers (dsRed, GCaMP6s, and CFP). Furthermore, single mosquito pairwise crosses were performed for at least 8 generations (Additional file 13: Figure S6C, D). To confirm homozygosity, single individuals starting from G8 were mated to wild-type. The resulting progeny was screened for 100% inheritance of the transgenic markers.The transgenic GCaMP6s/+/+ line has been deposited at BEI MR4 Resources (Accession # still waiting for acceptance of strain from BEI MR4).

### Genetics and molecular characterization of insertion site

To characterize the insertion site of GCaMP6s, we modified an inverse PCR protocol described previously [71, 81]. Briefly, genomic DNA(gDNA) was extracted from 10 *Ae. aegypti* fourth instar larvae using the DNeasy Blood & Tissue Kit (Qiagen, Hilden, Germany) in accordance with the manufacturer’s protocol. The eluted DNA was diluted, and two separate restriction digests were performed to characterize both the 5′ and 3′ ends using Sau3AI (5′ reaction) or HinP1I (3′ reaction) restriction enzymes. A ligation step using NEB T4 DNA Ligase was then performed on the restriction digest products to promote circularization of digested DNA. Two rounds of PCR were performed using primers 991.5F1, 991.5R1, 991.5F2, 991.5R2, 991.3F1, 991.3R1, 991.3F2 and 991.3R2 (with their corresponding restriction digest reaction) and sequence confirmation (1018) are listed in Additional file 16: Table S3. PCR products from the second round of PCR were cleaned using the MinElute PCR Purification Kit (Qiagen) in accordance with the manufacturer’s protocol, and subsequently sequenced by Sanger sequencing (Source BioScience). Both the location and orientation (chromosome 2, with the flanking genomic regions for the 5′ and 3′ *piggyBac* ends positioned at the genomic loci 285,175,805–285,176,289 and 285,175,275–285,175,803, respectively) were confirmed by PCR using primers designed from the mapped genomic region in combination with both 3′ *piggyBac* end forward primers. Sequencing data was then blasted to the AaegL5.0 reference genome. Alignment of the sequencing data was performed using EMBOSSWater (https://www.edi.ac.uk/Tools/psa/emboss_water/).

### Odor-evoked confocal imaging of non-water submerged larvae/adult

For larval imaging of GCaMP6s/+/+ calcium transients, using a slightly moistened fine tip paint brush, larva were placed ventral side down on double-sided tape adhered to a clean glass slide. Due to the larvae being exposed to air rather than its normal aquatic environment, to prevent dessication a moistened fine tip paint brush was used to periodically wet the larvae without affecting the sticky-tape adhesive. Imaging was focused on the full body and head. For adult imaging, mosquitoes were placed laterally on double-sided tape after being placed on ice for approximately 10 min. Antennae and proboscis were immobilized by using an artist brush and gently brushing the respective appendages onto the double sided tape. For both larvae and adults, a minimum of 15 s of inactivity was first captured recording the specimen. Recording continued for an additional 35 s. Images and recordings were taken using an Inverted Confocal microscope (Leica SP5).

### Odor-evoked confocal imaging of larvae in tethered-swimming assay

To immobilize each larval head, while allowing for movement of the larval body, less than one microliter of clear Aron Alpha high strength rapid bonding adhesive (Catalog # 72588) was applied to a Lab-Tek II chambered #1.5 German coverglass system composed of transparent borosilicate glass (Thermo Catalog #155382). Immediately following the application of the adhesive, the ventral side of a single larva was placed directly onto the adhesive, rapidly bonding the larval head to the coverglass in less than 1 min. The chamber was then filled with 500 µL of deionized water to fully submerge the larvae, while allowing for the larva’s respiratory siphon to meet the surface of the water. Before any recordings, the larvae was allowed to rest for 12 h to assimilate to the preparation. Recordings of stimulus-induced fluorescent responses were taken around the head. 100 µL of 5% solution of odorants were injected into the chamber after 15 s of inactivity in larval brains. Activity was measured from 15 s prior to addition of stimulus to 90 s after. Following each trial, stimuli were removed by draining the water in chamber, gently flushing the larvae and chamber three times, and refilling with fresh deionized water. The same larva was used for multiple stimulants.

### Selection and preparation of odorants

Stimulants were chosen from a list of known olfactory and/or gustatory stimuli of both *Drosophila melanogaster* and adult *Ae. aegypti* [26, 27, 36–41]. These included ethyl acetate (Sigma Cat# 319902), lactic acid (Sigma Cat# L1750), 1-octen-3-ol (Sigma Cat# O5284), butylamine (Sigma Cat# 471305), VUAA1 (Vitas-M Cat# STK047588), sucrose (Sigma Cat# S0389), lobeline (Sigma Cat# 141879), glutamate (Sigma Cat# 49621), and water (negative control). All stock solutions of odors were prepared as 5% solutions in water, with a final bioassayed concentration of 6 × 10^−5^ M. Food extract for larval experiments was prepared by mixing 0.5% fish food (Hikari Tropic First Bites: Petco, San Diego, CA, USA) in milliQ water. The solution was allowed to sit for 1 h, then filtered through a 0.2 μm sterile filter (#28145-477, VWR International, Radnor, PA, USA) to remove solid particulates. 1-octen-3-ol used in behavior experiments was prepared as a 10^−4^M solution in water, based on preliminary experiments testing several odor concentrations (data not shown).

### Imaging/data analyses

To quantify fluorescence responses to various stimuli, Leica LAS X Core Offline version 3.3.0 software was used to export raw fluorescence data from relevant ROIs. Further analysis was done using GraphPad Prism and RStudio. To account for differences in fluorescence intensity that differed between each larva, raw fluorescence was normalized using ΔF/F_0_ = (F − F_0_)/F_0_ where F is mean intensity of fluorescence at a certain time point and F_0_ is the baseline level of fluorescence using the average fluorescence intensity from the first 15 s of the recording without stimulation [82]. To determine the significance of responses to tested stimuli, a Welch’s t-test was conducted between the max ΔF/F_0_ of multiple replicates treated with one stimulant to that of water. Responses to each stimulant were compared to that of water. To compare the differences between GCaMP6s/+/+, GCaMP6s/orco5−/−, and GCaMP6s/Gr3−/− calcium responses to our simulus panel, a Welch’s t-test was conducted comparing the max ΔF/F_0_ values between two larval backgrounds in response to the same stimulus. Importantly, due to the methodology of our larval imaging assay, the larval abdomen would occasionally be viewable behind the ROI. To confirm that this interference does not create any significant artifacts while measuring raw fluorescence, raw fluorescence 2 s before and during interference were compared and no significant interference was detected (t = 0.237, p-value = 0.8158). Additionally, to confirm that our data was not confounded by differing base levels of expression due to differences between the wild-type and mutant strains, we performed an ANOVA comparing the average base levels of fluorescence measured prior to the addition of each stimulus between each strain. When compared, the mean expressions were not significantly different (p-value = 0.3212) hence background extraneous differences between out wild-type and mutant background strains may be negligible. In two-photon imaging experiments, a larva was transferred to a Peltier-cooled holder that allows for the head to be fixed to the stage using ultraviolet glue. GCaMP6s expression was imaged at 2 Hz using the Prairie Ultima IV two-photon excitation microscope (Prairie Technologies) and Ti–Sapphire laser (Chameleon Ultra; Coherent).

### Muscle/DE latency analysis

Temporal differences between muscle and DE responses were calculated by subtracting DE timepoints at 50% of maximum ΔF/F_0_ of the first peak following the addition of stimulus from that of muscles (Fig. 2b, c). Recordings with both DE and muscles not displaying clear peaks in response to stimulants as well as latency values greater than 15 were treated as NA. Latency values were converted into ordinal values of 4 categories: NA, negative, 0 (no difference), and positive. A Mann–Whitney U test between latency values from each stimulus and that of water was used to determine significant differences.

### Free-swimming larval behavior experiments

Larvae used for free-swimming behavior experiments were reared on Hikari Tropic First Bites (Petco, San Diego, CA, USA) under a 12 h light/dark cycle. One day before the experiment, 5-day old larvae were isolated into individual Falcon™ 50 mL conical centrifuge tubes (Thermo Fisher Scientific, Waltham, MA, USA) containing ~ 15 mL milliQ water and no food. During the experiment, individual larvae were introduced to the center of a dark behavior arena developed for assaying mosquito larval chemosensory preference (Fig. 4). No light was detected inside the arena under experimental conditions (LI-250A Lightmeter, instantaneous measurements, sensitive up to 0.01 µmol s^-1^ m^-2^ per µA. LI-COR Biosciences #Q40129). Animals were allowed to acclimate for 15 min in a custom 3D printed porcelain behavior chamber (ID #XWEEPACQA, Shapeways, New York, NY, USA) containing 20 mL of milliQ water. 100 µL of a chemical stimulus was then pipetted into the left side of the arena. Larvae were tested only during the day phase of the diurnal light cycle. Larvae were housed individually until eclosion to determine sex, and animals that died before eclosion were omitted from analyses.

Larval movement was recorded at 2fps for the 15 min acclimation period, as well as the 15 min experiment following stimulus introduction, using a Basler Scout Machine Vision Area Scan GigE camera (scA 1000-30gm, Ahrensburg, Germany) and Basler pylon Viewer Windows software. Larval trajectories were analyzed using ImageJ Fiji [83] and custom software written in Python (http://www.python.org): Multitracker by Floris van Breugel (https://github.com/florisvb/multi_tracker), as well as a batch-processing Multitracker add-on, Multivideo Multitracker by Eleanor Lutz (https://github.com/eleanorlutz/multivideo_multitracker) (Fig. 4). In brief, videos were cropped and contrast-enhanced in ImageJ Fiji. Larval position was extracted using frame-by-frame subtraction in Multitracker. Trajectories were manually inspected in the Multitracker GUI, where missing data points were added and extraneous tracked objects removed. We then converted trajectory position from pixel values to mm using the ratio of the known width of the behavior container. Finally, we calculated the instantaneous speed of the larva, in mm, for each frame. Using these position and speed values, we then calculated the mean instantaneous speed (mm/s) and preference index (PI; proportion of time spent in the odor half - proportion of time spent in the non-odor half).

### Fitness experiments

To determine the impact our GCaMP6s transgene insertion on mosquito fitness, a series of fitness experiments comparing the female fecundity, male fertility, larval hatchability, and duration between larval and pupal stages between our GCaMP6s +/+ line and the wild-type line the line was originally derived from. Female fecundity was determined by mating 100 virgin females of both the GCaMP6s +/+ and wild-type line to 50 wild-type males. Females were allowed to mate for 3 days after eclosion and were given access to anesthetized mice for 15 min on the 5th and 6th day after eclosion. Two days after blood feeding, single bloodfed females were individually captured into vials lined with moistened filter paper. Nonblood females were not collected. Bloodfed females were allowed 3 days in the vials to oviposit their eggs and were removed on the third day. Oviposited eggs were then counted. To determine male fertility, 25 males of both strains were mated to 100 virgin wild-type females and the same procedure for calculating female fecundity was used. To test egg hatching rate, eggs from single pair crosses of GCaMP6s +/+ (♀) X +/+ (♂) and +/+ (♀) X +/+ (♂) were counted and hatched 4 days after oviposited. Emerged larvae were then counted at the L2 stage. To calculate larvae to pupae development time, larvae of both strains were hatched and separate into 5 pans filled with 2.5L of water with 100 larvae per pan. The number of pupae emerged was counted everyday post hatching to estimate the number of days for larval developmental time.

### Immunofluorescence and GCaMP6s expression

To determine GCaMP6s’ pattern of expression, whole larval brains were stained following a previously published method [63]. Brains were either stained with a mixture of rabbit anti-GFP (1:500, abcam) and either mouse anti-alpha tubulin (1:100, DHSB) or mouse anti-GS (1:200, BD Bio) as primary antibodies. Secondary antibodies used were Alexa-488 donkey anti-rabbit and Alexa-555 donkey anti-mouse (ThermoFisher). Images of stained brains were taken using a Leica SP8 confocal microscope (Additional file 9: Figure S3). In addition, to examine the baseline fluorescence in different chemosensory cell types, processes (glia) and the lateral and medial cell bodies were recorded before and during odor stimulation and image planes were analyzed using k-means clustering. Based on this analysis, the ROI cell type clusters could be distinguished based on cell size alone, rather than differences in fluorescence (Additional file 6: Figure S2).

### Statistical analysis

To compare response differences between individuals that differed by either genetic background or stimulus, we used a Welch’s t-test due to the unequal sample size. To compare latency between neural and muscle responses, we used a Mann–Whitney U test which allows for analysis of non normalized distributions as well as ordinal data.A pairwise t-test was used to compare larval behavior during the acclimation phase of the experiment to the odor stimulation phase (Fig. 4). A 1-way ANOVA was used to compare overall behavioral differences across strains (Additional file 14: Figure S7).

## Additional files

**Additional file 1: Table S1.** Evaluating fitness cost of GCaMP6s insertion.

**Additional file 2: Video 1.** Time-lapse of female GCaMP6s/+/+ adult mouthparts taken on a Leica SP5 at 10 × magnification.

**Additional file 3: Figure S1.** GCaMP6s is a general tool to record calcium responses in multiple tissues. Calcium responses were imaged in multiple tissues (A-E, *left*) at varying developmental stages. Frames were taken every 9 s, starting at 0 s (i) until 36s (v) (A-E, *right*). Calcium transients were seen in regions within the adult female maxillary palp (ROI 1,4) and labium (ROI 2,3) (A). The female adult antennae also exhibited calcium transients within the nodes (ROI 1,2) and internodes (ROI 3) of the antennal flagellum (B). Stochastic patterns of fluorescence were seen when looking at clusters of ommatidia (ROI 1–3) within the right adult compound eye (C). Calcium transients were also visualized throughout the 2nd instar larvae. For example, responses were recorded in the medial retractor muscles (ROI 1), brain (ROI 2), longitudinal muscles in the thorax (ROI 3), longitudinal muscles in the 3rd abdominal segment (ROI 4), and muscles and neurons in the 6th abdominal segment (ROI 5) (D). When imaging the dorsal side of the larval head, calcium transients were visible in the transverse retractor (ROI 1), optic lobe (ROI 2), medial retractor (ROI 3), and antennal lobe (ROI 4) (E). Full videos have been provided in the supplement (Additional files 2: Video 1, 4: Video 2, 5: Video 3, 7: Video 4 and 8: Video 5).

**Additional file 4: Video 2.** Time-lapse of female GCaMP6s/+/+ adult antennae taken on a Leica SP5 at 10 × magnification.

**Additional file 5: Video 3.** Time-lapse of an adult female GCaMP6s/+/+ right compound eye on a Leica SP5 at 10 × magnification.

**Additional file 6: Figure S2.** GCaMP6s labeling is sufficient to morphologically distinguish various cell types of interest within the antennal lobe. GCaMP could be used to morphologically characterize diverse cell types including the volumes of individual glomeruli (A), (B) lateral cell cluster neurons (*B1*), volume of the PL2 glomerulus (*B2*), glia (*B3*), and medial cell cluster neurons (*B4*). (C) k-means clustering significantly identified (p < 0.05) three distinct cell classes based on their cell sizes, rather than differences in GCaMP expression levels, such as those in the lateral cell cluster (blue, possibly reflecting local interneurons), cells in the medial cell cluster (green, possibly projection neurons), and glial-like blebs on the glomerular surface (yellow). Cells were imaged at baseline levels and during odor stimulation (darker dots). Soma in the medial cell cluster exhibited greater changes in calcium dynamics during odor stimulation compared to the other cell types. Shaded areas denote the confidence interval around each cluster. (D) Responses of the different cell types during odor stimulation. Small, glia-like processes (yellow) showed small changes in calcium (less than 5%) compared to the soma in the medial (green) and lateral (blue) cell clusters. The projection neuron in the glomerulus (dashed green line) exhibited the largest calcium dynamic during odor stimulation.

**Additional file 7: Video 4.** Time-lapse of a whole L2 GCaMP6s/+/+ larvae taken on a Leica SP5 at 5 × magnification.

**Additional file 8: Video 5.** Time-lapse of a L2 GCaMP6s/+/+ larva head taken on a Leica SP5 at 10 × magnification.

**Additional file 9: Figure S3.** *PUb*-GCaMP6s pattern of expression within the mosquito larval brain. GCaMP6s +/+ larval brains were dissected, fixed and stained for GFP and either alpha-tubulin or glutamine synthetase (GS). Confocal imaging show colocalization between the respective neural or astrocyte-like glial cell antibodies with fixed GFP, demonstrating ubiquitous expression of GCaMP6s in both neural and glial cells.

**Additional file 10: Figure S4.** Calcium responses of GCaMP6s/+/+, GCaMP6s/orco5−/−, GCaMP6s/Gr3−/− to various stimulants. Time courses for GCaMP6s +/+, GCaMP6s/orco5−/−−/−, and GCaMP6s/Gr3−/− DE (purple) and muscle (blue) responses to a stimulus panel including 1-octen-3-ol, butylamine, ethyl acetate, lobeline, lactic acid, VUAA1, sucrose, glutamate, fish food, and water (control). The number of biological replicates used for each experiment were 3 or greater (GCaMP6s/+/+ DE: 1-octen-3-ol n = 6; butylamine n = 11; ethyl acetate n = 10; lobeline n = 8; lactic acid n = 6; VUAA1 n = 6; sucrose n = 8; glutamate n = 5; fish food n = 4; water n = 12. GCaMP6s/+/+ Muscle: 1-octen-3-ol n = 7; butylamine n = 15; ethyl acetate n = 15; lobeline n = 13; lactic acid n = 7; VUAA1 n = 8; sucrose n = 10; glutamate n = 7; fish food n = 3; water n = 12. GCaMP6s/orco5−/− DE: 1-octen-3-ol n = 4; butylamine n = 8; ethyl acetate n = 6; lobeline n = 6; lactic acid n = 6; VUAA1 n = 4; sucrose n = 7; glutamate n = 5; fish food n = 4; water n = 7. GCaMP6s/orco5−/− Muscle: 1-octen-3-ol n = 6; butylamine n = 10; ethyl acetate n = 6; lobeline n = 6; lactic acid n = 6; VUAA1 n = 5; sucrose n = 7; glutamate n = 5; fish food n = 4; water n = 9. GCaMP6s/Gr3−/− DE: 1-octen-3-ol n = 6; butylamine n = 5; ethyl acetate n = 5; lobeline n = 4; lactic acid n = 4; VUAA1 n = 6; sucrose n = 4; glutamate n = 4; fish food n = 6; water n = 4 GCaMP6s/Gr3−/− Muscle: 1-octen-3-ol n = 7; butylamine n = 11; ethyl acetate n = 7; lobeline n = 9; lactic acid n = 6; VUAA1 n = 8; sucrose n = 7; glutamate n = 5; fish food n = 5; water n = 10).

**Additional file 11: Figure S5.** Analysis of stimuli-evoked responses of GCaMP6s/+/+, GCaMP6s/orco5−/−, GCaMP6s/Gr3−/−. Maximum fluorescence values of the DE (purple) and Muscle (blue) in response to each stimulus was compared to that of water (control) to test for significance (A-C). Maximum changes in fluorescence in response to each stimulus was also compared between the DE and muscles of GCaMP6s/+/+ (green) and both GCaMP6s/orco5−/− (red) and GCaMP6s/Gr3−/− (blue) (D, E). The number of biological replicates used for each experiment were 3 or greater. *: p-value < 0.05, **: p-value < 0.01, ***: p-value < 0.001, Welch’s T-test (GCaMP6s/+/+ DE: 1-octen-3-ol n = 6; butylamine n = 11; ethyl acetate n = 10; lobeline n = 8; lactic acid n = 6; VUAA1 n = 6; sucrose n = 8; glutamate n = 5; fish food n = 4; water n = 12. GCaMP6s/+/+ Muscle: 1-octen-3-ol n = 7; butylamine n = 15; ethyl acetate n = 15; lobeline n = 13; lactic acid n = 7; VUAA1 n = 8; sucrose n = 10; glutamate n = 7; fish food n = 3; water n = 12. GCaMP6s/orco5−/− DE: 1-octen-3-ol n = 4; butylamine n = 8; ethyl acetate n = 6; lobeline n = 6; lactic acid n = 6; VUAA1 n = 4; sucrose n = 7; glutamate n = 5; fish food n = 4; water n = 7. GCaMP6s/orco5−/− Muscle: 1-octen-3-ol n = 6; butylamine n = 10; ethyl acetate n = 6; lobeline n = 6; lactic acid n = 6; VUAA1 n = 5; sucrose n = 7; glutamate n = 5; fish food n = 4; water n = 9. GCaMP6s/Gr3−/− DE: 1-octen-3-ol n = 6; butylamine n = 5; ethyl acetate n = 5; lobeline n = 4; lactic acid n = 4; VUAA1 n = 6; sucrose n = 4; glutamate n = 4; fish food n = 6; water n = 4 GCaMP6s/Gr3−/− Muscle: 1-octen-3-ol n = 7; butylamine n = 11; ethyl acetate n = 7; lobeline n = 9; lactic acid n = 6; VUAA1 n = 8; sucrose n = 7; glutamate n = 5; fish food n = 5; water n = 10).

**Additional file 12: Figure S8.** Average calcium responses of GCaMP6s +/+, GCaMP/orco5−/−, and GCaMP6s/Gr3−/− over time. Responses to various stimuli were averaged over multiple replicates for each time point.(GCaMP6s/+/+ DE: 1-octen-3-ol n = 6; butylamine n = 11; ethyl acetate n = 1015; lobeline n = 813; lactic acid n = 67; VUAA1 n = 68; sucrose n = 810; glutamate n = 57; fish food n = 43; water n = 12. GCaMP6s/+/+ Muscle: 1-octen-3-ol n = 7; butylamine n = 15; ethyl acetate n = 15; lobeline n = 13; lactic acid n = 7; VUAA1 n = 8; sucrose n = 10; glutamate n = 7; fish food n = 3; water n = 12. GCaMP6s/orco5−/− DE: 1-octen-3-ol n = 46; butylamine n = 811; ethyl acetate n = 6; lobeline n = 6; lactic acid n = 6; VUAA1 n = 45; sucrose n = 7; glutamate n = 5; fish food n = 4; water n = 712. GCaMP6s/orco5−/− Muscle: 1-octen-3-ol n = 6; butylamine n = 10; ethyl acetate n = 6; lobeline n = 6; lactic acid n = 6; VUAA1 n = 5; sucrose n = 7; glutamate n = 5; fish food n = 4; water n = 9. GCaMP6s/Gr3−/− DE: 1-octen-3-ol n = 6; butylamine n = 511; ethyl acetate n = 57; lobeline n = 49; lactic acid n = 46; VUAA1 n = 68; sucrose n = 47; glutamate n = 45; fish food n = 65; water n = 412 GCaMP6s/Gr3−/− Muscle: 1-octen-3-ol n = 7; butylamine n = 11; ethyl acetate n = 7; lobeline n = 9; lactic acid n = 6; VUAA1 n = 8; sucrose n = 7; glutamate n = 5; fish food n = 5; water n = 10).

**Additional file 13: Figure S6.** GCaMP6s/orco5−/− and GCaMP6s/Gr3−/− Line Generation and Confirmation. GCaMP6s +/+ mosquitos were mated to orco5−/− mosquitoes. Resulting individuals were mated to orco5−/− mosquitoes in a single pairwise cross for at least 8 generations and sequenced using Sanger Sequencing to confirm the presence of the *orco* gene (A). Mutations are indicated in red (B). GCaMP6s/+/+ mosquitos were crossed with Gr3−/− mosquitos. Individuals containing both GCaMP6s and Gr3−/− markers, dsRed/GFP transients and CFP respectively, were crossed in single pairwise matings for at least 8 generations to generate homozygous lines, individuals were then crossed to +/+ to confirm homozygosity by mendelian inheritance (C). All mosquito lines used were screened and sorted during the larval stage using a longpass-GFP and CFP filter to confirm OpIE-DsRed/GCaMP and CFP respectively (D).

**Additional file 14: Figure S7:** Larvae of different strains do not exhibit motility defects Prior to stimulation we found no differences in positional preference (A) or mean speed (B) between larvae of the mutant and wild-type backgrounds (1-way ANOVA by background, p > 0.05). Our results suggest that our arena is fair in the absence of odors (A) and that larvae of different strains do not exhibit motility defects (B). Gray bars show mean ± SEM. n = 14 ~ 24 per treatment (GCaMP6s/+/+: water n = 20, 1-octen-3-ol n = 14, food extract n = 20. GCaMP6s/orco5−/−: water n = 24, 1-octen-3-ol n = 16, food extract n = 20. GCaMP6s/Gr3−/−: water n = 16, 1-octen-3-ol n = 17, food extract n = 16, Liverpool wt: water n = 19).

**Additional file 15: Table S2.** Primer Sequences used in this study.

**Additional file 16: Table S3.** Inverse PCR Primer sequences used in this study.

## Data Availability

The datasets and videos used and/or analysed during the current study are available from the corresponding author on reasonable request. The Python code used to analyze and interpret larval behavior assays, as well as raw trajectory data for each larval behavior experiment (Fig. 4, Additional file 14: Figure S7) are available at https://github.com/eleanorlutz/aedes-aegypti-gcamp6s-larval-behavior.

## Abbreviations

RIDL: release of insects carrying dominant lethal
GECI: Genetically Encoded Calcium Indicator
*PUb*: *polyubiquitin* promoter (AAEL003877)
ROI: regions-of-interest
DE: deuterocerebrum

## References

[1] Weaver SC, Charlier C, Vasilakis N, Lecuit M (2017). Zika, Chikungunya, and other emerging vector-borne viral diseases. Annu Rev Med.

[2] Barrett ADT, Higgs S (2007). Yellow fever: a disease that has yet to be conquered. Annu Rev Entomol.

[3] Halstead SB (2008). Dengue virus-mosquito interactions. Annu Rev Entomol.

[4] Weaver SC, Barrett ADT (2004). Transmission cycles, host range, evolution and emergence of arboviral disease. Nat Rev Microbiol.

[5] Weaver SC, Reisen WK (2010). Present and future arboviral threats. Antiviral Res.

[6] Harris AF, McKemey AR, Nimmo D, Curtis Z, Black I, Morgan SA (2012). Successful suppression of a field mosquito population by sustained release of engineered male mosquitoes. Nat Biotechnol.

[7] McMeniman CJ, Lane RV, Cass BN, Fong AWC, Sidhu M, Wang Y-F (2009). Stable introduction of a life-shortening Wolbachia infection into the mosquito *Aedes aegypti*. Science.

[8] Walker T, Johnson PH, Moreira LA, Iturbe-Ormaetxe I, Frentiu FD, McMeniman CJ (2011). The wMel Wolbachia strain blocks dengue and invades caged *Aedes aegypti* populations. Nature.

[9] Sinkins SP (2004). Wolbachia and cytoplasmic incompatibility in mosquitoes. Insect Biochem Mol Biol.

[10] Champer J, Buchman A, Akbari OS (2016). Cheating evolution: engineering gene drives to manipulate the fate of wild populations. Nat Rev Genet.

[11] Marshall JM, Akbari OS (2018). Can CRISPR-based gene drive be confined in the wild? A question for molecular and population biology. ACS Chem Biol.

[12] Marshall John M., Akbari Omar S. (2016). Gene Drive Strategies for Population Replacement. Genetic Control of Malaria and Dengue.

[13] Kandul NP, Liu J, Sanchez HM, Wu SL, Marshall JM, Akbari OS (2018). Transforming insect population control with precision guided sterile males.. BioRxiv.

[14] Li M, Yang T, Kandul NP, Bui M, Gamez S, Raban R, et al. Development of a Confinable Gene-Drive System in the Human Disease Vector, *Aedes aegypti*. 2019. 10.1101/645440. 10.7554/eLife.51701 PMC6974361 31960794

[15] Maoz D, Ward T, Samuel M, Müller P, Runge-Ranzinger S, Toledo J (2017). Community effectiveness of pyriproxyfen as a dengue vector control method: a systematic review. PLoS Negl Trop Dis..

[16] Moyes CL, Vontas J, Martins AJ, Ng LC, Koou SY, Dusfour I (2017). Contemporary status of insecticide resistance in the major Aedes vectors of arboviruses infecting humans. PLoS Negl Trop Dis..

[17] Franz AWE, Clem RJ, Passarelli AL (2014). Novel genetic and molecular tools for the investigation and control of dengue virus transmission by mosquitoes. Curr Trop Med Rep..

[18] Montell C, Zwiebel LJ, Raikhel AS (2016). Chapter ten—mosquito sensory systems. Advances in insect physiology.

[19] Lutz EK, Lahondère C, Vinauger C, Riffell JA (2017). Olfactory learning and chemical ecology of olfaction in disease vector mosquitoes: a life history perspective. Curr Opin Insect Sci..

[20] Syed Z (2015). Chemical ecology and olfaction in arthropod vectors of diseases. Curr Opin Insect Sci..

[21] DeGennaro M, McBride CS, Seeholzer L, Nakagawa T, Dennis EJ, Goldman C (2013). orco mutant mosquitoes lose strong preference for humans and are not repelled by volatile DEET. Nature.

[22] McMeniman CJ, Corfas RA, Matthews BJ, Ritchie SA, Vosshall LB (2014). Multimodal integration of carbon dioxide and other sensory cues drives mosquito attraction to humans. Cell.

[23] van Breugel F, Riffell J, Fairhall A, Dickinson MH (2015). Mosquitoes use vision to associate odor plumes with thermal targets. Curr Biol.

[24] Kennedy JS (2009). The visual responses of flying mosquitoes. Proc Zool Soc Lond..

[25] Vinauger C, Lahondère C, Wolff GH, Locke LT, Liaw JE, Parrish JZ (2018). Modulation of host learning in *Aedes aegypti* mosquitoes. Curr Biol.

[26] Xia Y, Wang G, Buscariollo D, Pitts RJ, Wenger H, Zwiebel LJ (2008). The molecular and cellular basis of olfactory-driven behavior in *Anopheles gambiae* larvae. Proc Natl Acad Sci USA.

[27] Liu C, Jason Pitts R, Bohbot JD, Jones PL, Wang G, Zwiebel LJ (2010). Distinct olfactory signaling mechanisms in the malaria vector mosquito *Anopheles gambiae*. PLoS Biol.

[28] Christophers S. *Aedes aegypti* (L.) the yellow fever mosquito: its life history, bionomics and structure. aegypti (L) the Yellow Fever Mosquito: its Life …. 1960. https://www.cabdirect.org/cabdirect/abstract/19602901825.

[29] Ghaninia M, Larsson M, Hansson BS, Ignell R (2008). Natural odor ligands for olfactory receptor neurons of the female mosquito *Aedes aegypti*: use of gas chromatography-linked single sensillum recordings. J Exp Biol.

[30] Moffett DF, Jagadeshwaran U, Wang Z, Davis HM, Onken H, Goss GG (2012). Signaling by intracellular Ca2 and H in larval mosquito (*Aedes aegypti*) midgut epithelium in response to serosal serotonin and lumen pH. J Insect Physiol.

[31] Macleod GT, Hegström-Wojtowicz M, Charlton MP, Atwood HL (2002). Fast calcium signals in Drosophila motor neuron terminals. J Neurophysiol.

[32] Chen T-W, Wardill TJ, Sun Y, Pulver SR, Renninger SL, Baohan A (2013). Ultrasensitive fluorescent proteins for imaging neuronal activity. Nature.

[33] Anderson MAE, Gross TL, Myles KM, Adelman ZN (2010). Validation of novel promoter sequences derived from two endogenous ubiquitin genes in transgenic *Aedes aegypti*. Insect Mol Biol.

[34] Akbari OS, Antoshechkin I, Amrhein H, Williams B, Diloreto R, Sandler J (2013). The developmental transcriptome of the mosquito *Aedes aegypti*, an invasive species and major arbovirus vector. Graph..

[35] Mysore K, Flister S, Müller P, Rodrigues V, Reichert H (2011). Brain development in the yellow fever mosquito *Aedes aegypti*: a comparative immunocytochemical analysis using cross-reacting antibodies from Drosophila melanogaster. Dev Genes Evol.

[36] Taylor RW, Romaine IM, Liu C, Murthi P, Jones PL, Waterson AG (2012). Structure-activity relationship of a broad-spectrum insect odorant receptor agonist. ACS Chem Biol.

[37] Reddy GVP, Guerrero A (2004). Interactions of insect pheromones and plant semiochemicals. Trends Plant Sci.

[38] Acree F, Turner RB, Gouck HK, Beroza M, Smith N (1968). L-lactic acid: a mosquito attractant isolated from humans. Science.

[39] Hoel DF, Kline DL, Allan SA, Grant A (2007). Evaluation of carbon dioxide, 1-octen-3-ol, and lactic acid as baits in Mosquito Magnet Pro traps for Aedes albopictus in north central Florida. J Am Mosq Control Assoc..

[40] Gonzalez PV, González Audino PA, Masuh HM (2015). Behavioral response of *Aedes aegypti* (Diptera: Culicidae) larvae to synthetic and natural attractants and repellents. J Med Entomol.

[41] Freeman EG, Wisotsky Z, Dahanukar A (2014). Detection of sweet tastants by a conserved group of insect gustatory receptors. Proc Natl Acad Sci USA.

[42] Montell C (2009). A taste of the Drosophila gustatory receptors. Curr Opin Neurobiol.

[43] Chitarra GS, Abee T, Rombouts FM, Posthumus MA, Dijksterhuis J (2004). Germination of penicillium paneum Conidia is regulated by 1-octen-3-ol, a volatile self-inhibitor. Appl Environ Microbiol.

[44] Broek IVF, Otter CJ (2000). Odour sensitivity of antennal olfactory cells underlying grooved pegs of *Anopheles gambiae* quadriannulatus. Entomol Exp Appl.

[45] Kumar BN, Taylor RW, Pask GM, Zwiebel LJ, Newcomb RD, Christie DL (2013). A conserved aspartic acid is important for agonist (VUAA1) and odorant/tuning receptor-dependent activation of the insect odorant co-receptor (Orco). PLoS ONE.

[46] Bohbot JD, Dickens JC (2009). Characterization of an enantioselective odorant receptor in the yellow fever mosquito *Aedes aegypti*. PLoS ONE.

[47] Erdelyan CNG, Mahood TH, Bader TSY, Whyard S (2012). Functional validation of the carbon dioxide receptor genes in *Aedes aegypti* mosquitoes using RNA interference. Insect Mol Biol.

[48] Bohbot J, Pitts RJ, Kwon H-W, Rützler M, Robertson HM, Zwiebel LJ (2007). Molecular characterization of the *Aedes aegypti* odorant receptor gene family. Insect Mol Biol.

[49] Merritt RW, Dadd RH, Walker ED (1992). Feeding behavior, natural food, and nutritional relationships of larval mosquitoes. Annu Rev Entomol.

[50] Riffell JA, Abrell L, Hildebrand JG (2008). Physical processes and real-time chemical measurement of the insect olfactory environment. J Chem Ecol.

[51] Burks RL, Lodge DM (2002). Cued advances and opportunities in freshwater chemical ecology. J Chem Ecol.

[52] Mysore K, Flannery EM, Tomchaney M, Severson DW, Duman-Scheel M (2013). Disruption of *Aedes aegypti* olfactory system development through chitosan/siRNA nanoparticle targeting of semaphorin-1a. PLoS Negl Trop Dis..

[53] Crespo JG (2011). A review of chemosensation and related behavior in aquatic insects. J Insect Sci..

[54] Mellon D (2007). Combining dissimilar senses: central processing of hydrodynamic and chemosensory inputs in aquatic crustaceans. Biol Bull.

[55] Thiel Martin, Breithaupt Thomas (2010). Chemical Communication in Crustaceans: Research Challenges for the Twenty-First Century. Chemical Communication in Crustaceans.

[56] Takken W, Kline DL (1989). Carbon dioxide and 1-octen-3-ol as mosquito attractants. J Am Mosq Control Assoc..

[57] Fischer G, Dott W (2003). Relevance of airborne fungi and their secondary metabolites for environmental, occupational and indoor hygiene. Arch Microbiol.

[58] Gomez-Marin A, Stephens GJ, Louis M (2011). Active sampling and decision making in Drosophila chemotaxis. Nat Commun..

[59] Sourjik V, Wingreen NS (2012). Responding to chemical gradients: bacterial chemotaxis. Curr Opin Cell Biol.

[60] Hilliard MA, Bargmann CI, Bazzicalupo PC (2002). elegans responds to chemical repellents by integrating sensory inputs from the head and the tail. Curr Biol.

[61] Bräcker LB, Siju KP, Varela N, Aso Y, Zhang M, Hein I (2013). Essential role of the mushroom body in context-dependent CO_2_ avoidance in Drosophila. Curr Biol.

[62] Lewis LPC, Siju KP, Aso Y, Friedrich AB, Bulteel AJB, Rubin GM (2015). A higher brain circuit for immediate integration of conflicting sensory information in Drosophila. Curr Biol.

[63] Riabinina O, Task D, Marr E, Lin C-C, Alford R, O’Brochta DA (2016). Organization of olfactory centres in the malaria mosquito *Anopheles gambiae*. Nat Commun..

[64] Chen C-L, Hermans L, Viswanathan MC, Fortun D, Unser M, Cammarato A, et al. Imaging neural activity in the ventral nerve cord of behaving adult Drosophila. bioRxiv. 2018;250118. 10.1101/250118. 10.1038/s41467-018-06857-z PMC6197219 30348941

[65] Schnell B, Ros IG, Dickinson MH (2017). A descending neuron correlated with the rapid steering maneuvers of flying Drosophila. Curr Biol.

[66] Kim AJ, Fenk LM, Lyu C, Maimon G (2017). Quantitative predictions orchestrate visual signaling in Drosophila. Cell.

[67] Namiki S, Dickinson MH, Wong AM, Korff W, Card GM. The functional organization of descending sensory-motor pathways in Drosophila. 2017. 10.1101/231696. 10.7554/eLife.34272 PMC6019073 29943730

[68] Lindsay T, Sustar A, Dickinson M (2017). The function and organization of the motor system controlling flight maneuvers in flies. Curr Biol.

[69] Melis JM, Lindsay T, Dickinson MH (2018). Mapping steering muscle activity to 3-dimensional wing kinematics in fruit flies. Integr Comp Biol.

[70] Pelletier J, Guidolin A, Syed Z, Cornel AJ, Leal WS (2010). Knockdown of a mosquito odorant-binding protein involved in the sensitive detection of oviposition attractants. J Chem Ecol.

[71] Li M, Bui M, Yang T, Bowman CS, White BJ, Akbari OS (2017). Germline Cas9 expression yields highly efficient genome engineering in a major worldwide disease vector, *Aedes aegypti*. Proc Natl Acad Sci USA.

[72] Gibson DG, Young L, Chuang R-Y, Craig Venter J, Hutchison CA, Smith HO (2009). Enzymatic assembly of DNA molecules up to several hundred kilobases. Nat Methods.

[73] Bischof J, Maeda RK, Hediger M, Karch F, Basler K (2007). An optimized transgenesis system for Drosophila using germ-line-specific φC31 integrases. Proc Natl Acad Sci.

[74] Pfeiffer BD, Truman JW, Rubin GM (2012). Using translational enhancers to increase transgene expression in Drosophila. Proc Natl Acad Sci USA.

[75] Theilmann DA, Stewart S (1992). Tandemly repeated sequence at the 3′ end of the IE-2 gene of the baculovirus Orgyia pseudotsugata multicapsid nuclear polyhedrosis virus is an enhancer element. Virology.

[76] Handler A. M., Harrell Ii R. A. (1999). Germline transformation of Drosophila melanogaster with the piggyBac transposon vector. Insect Molecular Biology.

[77] Kokoza V, Ahmed A, Wimmer EA, Raikhel AS (2001). Efficient transformation of the yellow fever mosquito *Aedes aegypti* using the piggyBac transposable element vector pBac[3xP3-EGFP afm]. Insect Biochem Mol Biol.

[78] Lobo NF, Hua-Van A, Li X, Nolen BM, Fraser MJ (2002). Germ line transformation of the yellow fever mosquito, *Aedes aegypti*, mediated by transpositional insertion of a piggyBac vector. Insect Mol Biol.

[79] Akbari OS, Papathanos PA, Sandler JE, Kennedy K, Hay BA (2014). Identification of germline transcriptional regulatory elements in *Aedes aegypti*. Sci Rep..

[80] Aryan A, Myles KM, Adelman ZN (2014). Targeted genome editing in *Aedes aegypti* using TALENs. Methods.

[81] Huang A. M., Rehm E. J., Rubin G. M. (2009). Recovery of DNA Sequences Flanking P-Element Insertions in Drosophila: Inverse PCR and Plasmid Rescue. Cold Spring Harbor Protocols.

[82] Jia H, Rochefort NL, Chen X, Konnerth A (2011). In vivo two-photon imaging of sensory-evoked dendritic calcium signals in cortical neurons. Nat Protoc.

[83] Schindelin J, Arganda-Carreras I, Frise E, Kaynig V, Longair M, Pietzsch T (2012). Fiji: an open-source platform for biological-image analysis. Nat Methods.

