# Supplementary material for: Live calcium imaging of Aedes aegypti neuronal tissues reveals differential importance of chemosensory systems for life-history-specific foraging strategies
Source: BMC Neurosci. 2019 Jun 17;20:27. doi: 10.1186/s12868-019-0511-y (PMC6580577; doi:10.1186/s12868-019-0511-y)
Supplement: Supplementary file 1 — Additional file 1: Table S1. Evaluating fitness cost of GCaMP6s insertion. [file 12868_2019_511_MOESM1_ESM.docx]

**Additional file 1: Table S1. Evaluating fitness cost of GCaMP6s insertion.**

|  | GCaMP6s+/+ | WT+/+ | p-value |
| --- | --- | --- | --- |
| Fertility (# eggs laid) | 76.42±18.97 (n=59) | 79.23±21.65 (n=74) | 0.4276 |
| Fecundity (# eggs laid) | 96.08±30.29 (n=72) | 79.23±21.65 (n=74) | >0.05 |
| Egg hatching rate (% eggs hatched) | 64.48±32.56 (n=72) | 71.78±28.57(n=74) | 0.1536 |
| Larval development time (days) | 6.21±0.82 (n=434) | 6.27±0.49 (n=516) | 0.2034 |
