# Supplementary material for: Live calcium imaging of Aedes aegypti neuronal tissues reveals differential importance of chemosensory systems for life-history-specific foraging strategies
Source: BMC Neurosci. 2019 Jun 17;20:27. doi: 10.1186/s12868-019-0511-y (PMC6580577; doi:10.1186/s12868-019-0511-y)
Supplement: Supplementary file 3 — Additional file 3: Figure S1. GCaMP6s is a general tool to record calcium responses in multiple tissues. Calcium responses were imaged in multiple tissues (A-E, left) at varying developmental stages. Frames were taken every 9 s, starting at 0 s (i) until 36s (v) (A-E, right). Calcium transients were seen in regions within the adult female maxillary palp (ROI 1,4) and labium (ROI 2,3) (A). The female adult antennae also exhibited calcium transients within the nodes (ROI 1,2) and internodes (ROI 3) of the antennal flagellum (B). Stochastic patterns of fluorescence were seen when looking at clusters of ommatidia (ROI 1–3) within the right adult compound eye (C). Calcium transients were also visualized throughout the 2nd instar larvae. For example, responses were recorded in the medial retractor muscles (ROI 1), brain (ROI 2), longitudinal muscles in the thorax (ROI 3), longitudinal muscles in the 3rd abdominal segment (ROI 4), and muscles and neurons in the 6th abdominal segment (ROI 5) (D). When imaging the dorsal side of the larval head, calcium transients were visible in the transverse retractor (ROI 1), optic lobe (ROI 2), medial retractor (ROI 3), and antennal lobe (ROI 4) (E). Full videos have been provided in the supplement (Additional files 2: Video 1, 4: Video 2, 5: Video 3, 7: Video 4 and 8: Video 5). [file 12868_2019_511_MOESM3_ESM.docx]

**
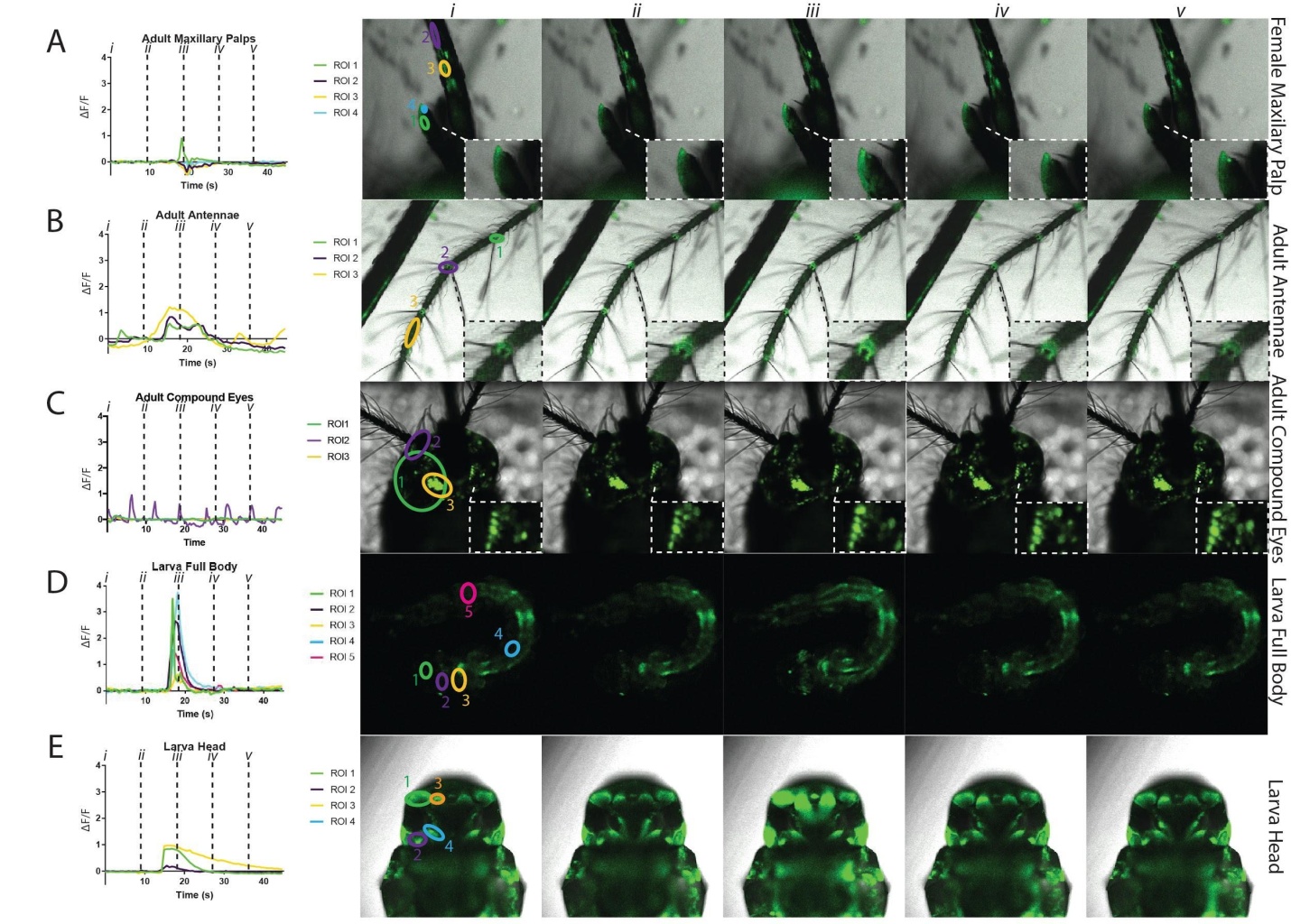
**

**Additional file 3: Figure S1. GCaMP6s is a general tool to record calcium responses in multiple tissues.**

Calcium responses were imaged in multiple tissues (A-E, *left*) at varying developmental stages. Frames were taken every 9s, starting at 0s (i) until 36s (v) (A-E, *right*). Calcium transients were seen in regions within the adult female maxillary palp (ROI 1,4) and labium (ROI 2,3) (A). The female adult antennae also exhibited calcium transients within the nodes (ROI 1,2) and internodes (ROI 3) of the antennal flagellum (B). Stochastic patterns of fluorescence were seen when looking at clusters of ommatidia (ROI 1-3) within the right adult compound eye (C). Calcium transients were also visualized throughout the 2nd instar larvae. For example, responses were recorded in the medial retractor muscles (ROI 1), brain (ROI 2), longitudinal muscles in the thorax (ROI 3), longitudinal muscles in the 3rd abdominal segment (ROI 4), and muscles and neurons in the 6th abdominal segment (ROI 5) (D). When imaging the dorsal side of the larval head, calcium transients were visible in the transverse retractor (ROI 1), optic lobe (ROI 2), medial retractor (ROI 3), and antennal lobe (ROI 4) (E). Full videos have been provided in the supplement (Supplementary videos 1-5).
