# Supplementary material for: Live calcium imaging of Aedes aegypti neuronal tissues reveals differential importance of chemosensory systems for life-history-specific foraging strategies
Source: BMC Neurosci. 2019 Jun 17;20:27. doi: 10.1186/s12868-019-0511-y (PMC6580577; doi:10.1186/s12868-019-0511-y)
Supplement: Supplementary file 6 — Additional file 6: Figure S2. GCaMP6s labeling is sufficient to morphologically distinguish various cell types of interest within the antennal lobe. GCaMP could be used to morphologically characterize diverse cell types including the volumes of individual glomeruli (A), (B) lateral cell cluster neurons (B1), volume of the PL2 glomerulus (B2), glia (B3), and medial cell cluster neurons (B4). (C) k-means clustering significantly identified (p < 0.05) three distinct cell classes based on their cell sizes, rather than differences in GCaMP expression levels, such as those in the lateral cell cluster (blue, possibly reflecting local interneurons), cells in the medial cell cluster (green, possibly projection neurons), and glial-like blebs on the glomerular surface (yellow). Cells were imaged at baseline levels and during odor stimulation (darker dots). Soma in the medial cell cluster exhibited greater changes in calcium dynamics during odor stimulation compared to the other cell types. Shaded areas denote the confidence interval around each cluster. (D) Responses of the different cell types during odor stimulation. Small, glia-like processes (yellow) showed small changes in calcium (less than 5%) compared to the soma in the medial (green) and lateral (blue) cell clusters. The projection neuron in the glomerulus (dashed green line) exhibited the largest calcium dynamic during odor stimulation. [file 12868_2019_511_MOESM6_ESM.docx]

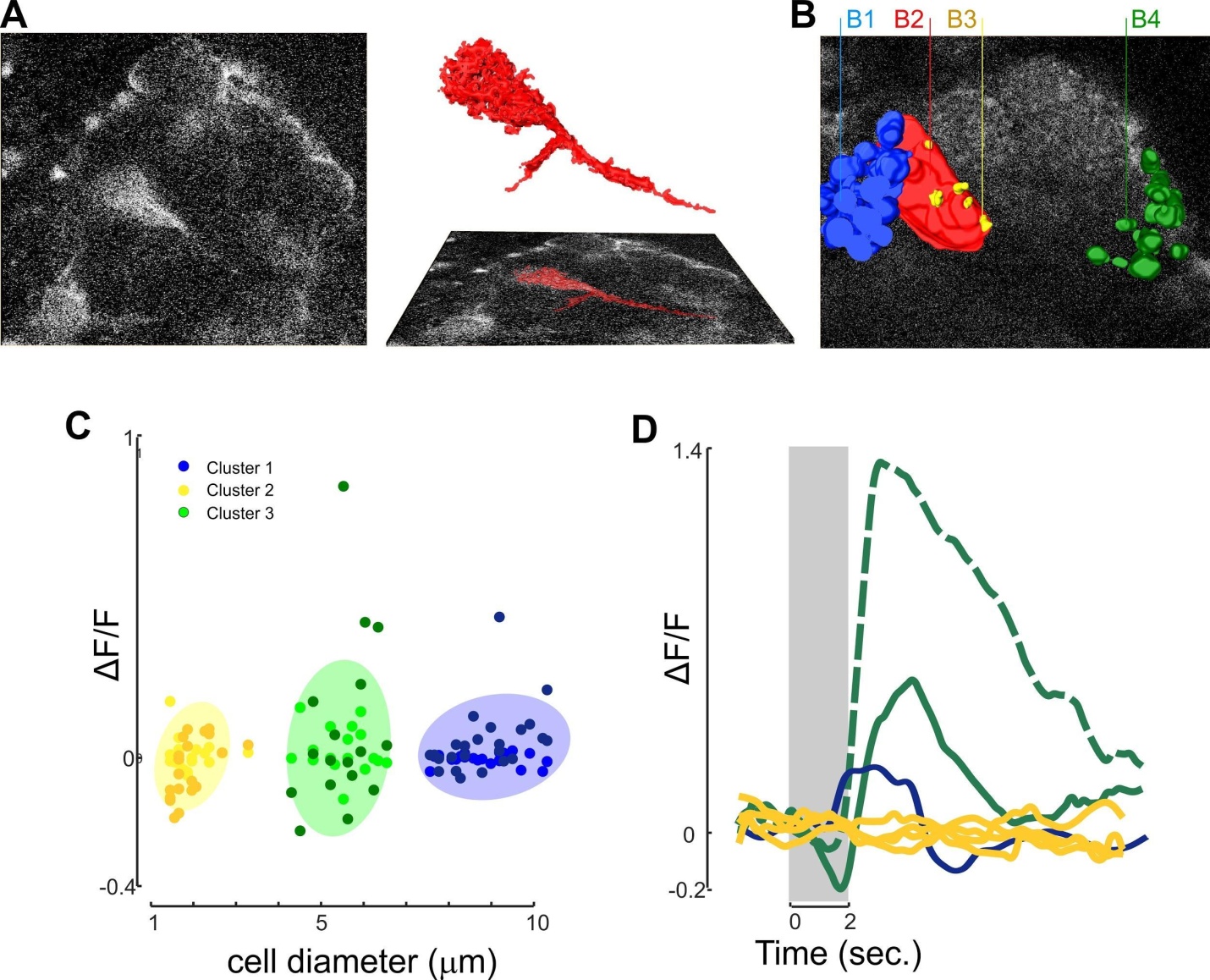


**Additional file 6: Figure S2. GCaMP6s labeling is sufficient to morphologically distinguish various cell types of interest within the antennal lobe.** GCaMP could be used to morphologically characterize diverse cell types including the volumes of individual glomeruli (A), (B) lateral cell cluster neurons (*B1*), volume of the PL2 glomerulus (*B2*), glia (*B3*), and medial cell cluster neurons (*B4*). (C) k-means clustering significantly identified (p<0.05) three distinct cell classes based on their cell sizes, rather than differences in GCaMP expression levels, such as those in the lateral cell cluster (blue, possibly reflecting local interneurons), cells in the medial cell cluster (green, possibly projection neurons), and glial-like blebs on the glomerular surface (yellow). Cells were imaged at baseline levels and during odor stimulation (darker dots). Soma in the medial cell cluster exhibited greater changes in calcium dynamics during odor stimulation compared to the other cell types. Shaded areas denote the confidence interval around each cluster. (D) Responses of the different cell types during odor stimulation. Small, glia-like processes (yellow) showed small changes in calcium (less than 5%) compared to the soma in the medial (green) and lateral (blue) cell clusters. The projection neuron in the glomerulus (dashed green line) exhibited the largest calcium dynamic during odor stimulation.
