# Supplementary material for: Live calcium imaging of Aedes aegypti neuronal tissues reveals differential importance of chemosensory systems for life-history-specific foraging strategies
Source: BMC Neurosci. 2019 Jun 17;20:27. doi: 10.1186/s12868-019-0511-y (PMC6580577; doi:10.1186/s12868-019-0511-y)
Supplement: Supplementary file 9 — Additional file 9: Figure S3. PUb-GCaMP6s pattern of expression within the mosquito larval brain. GCaMP6s +/+ larval brains were dissected, fixed and stained for GFP and either alpha-tubulin or glutamine synthetase (GS). Confocal imaging show colocalization between the respective neural or astrocyte-like glial cell antibodies with fixed GFP, demonstrating ubiquitous expression of GCaMP6s in both neural and glial cells. [file 12868_2019_511_MOESM9_ESM.docx]

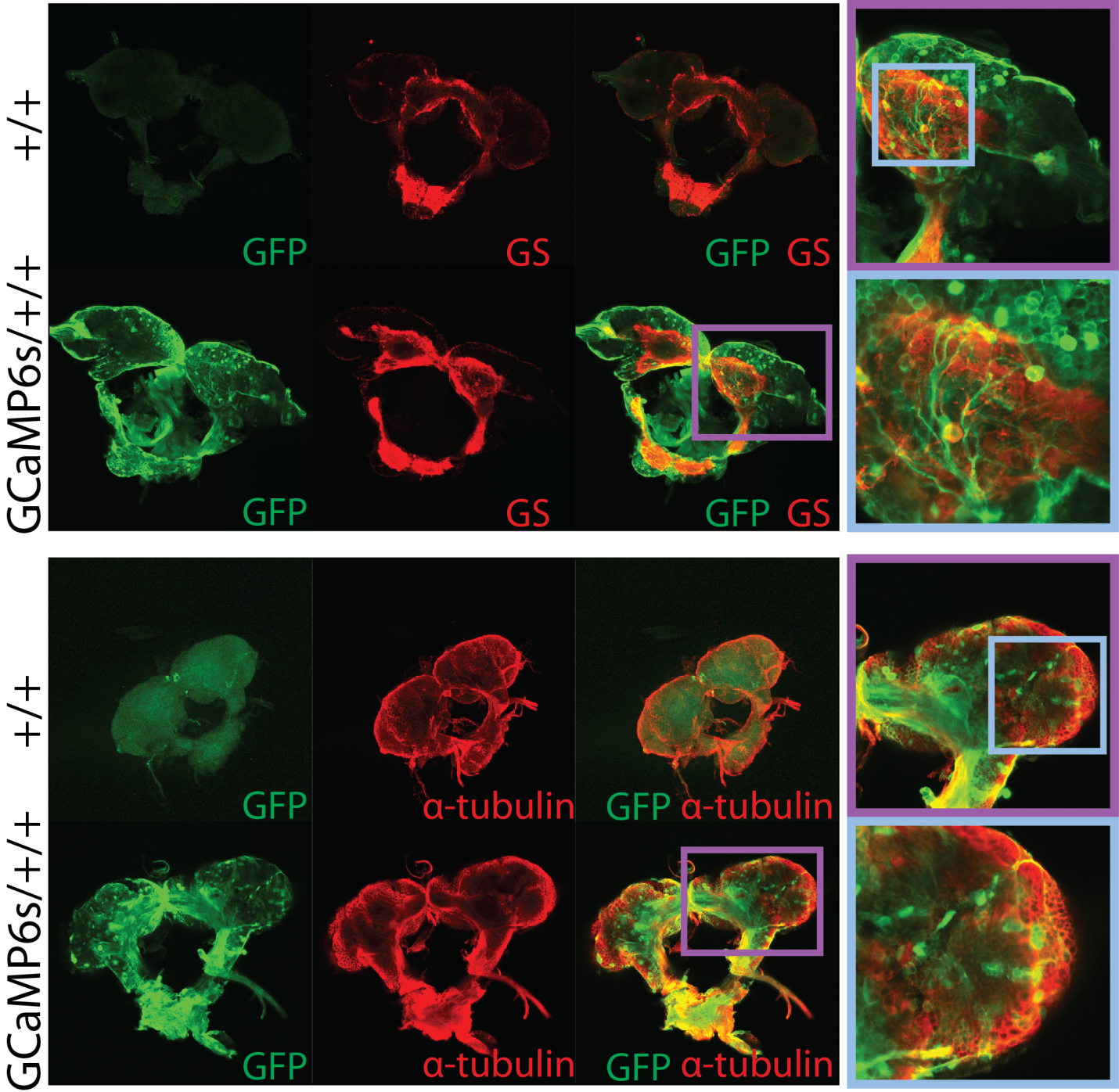


**Additional file 9: Figure S3. *PUb*-GCaMP6s pattern of expression within the mosquito larval brain.** GCaMP6s+/+ larval brains were dissected, fixed and stained for GFP and either alpha-tubulin or glutamine synthetase (GS). Confocal imaging show colocalization between the respective neural or astrocyte-like glial cell antibodies with fixed GFP, demonstrating ubiquitous expression of GCaMP6s in both neural and glial cells.
