# Supplementary material for: Live calcium imaging of Aedes aegypti neuronal tissues reveals differential importance of chemosensory systems for life-history-specific foraging strategies
Source: BMC Neurosci. 2019 Jun 17;20:27. doi: 10.1186/s12868-019-0511-y (PMC6580577; doi:10.1186/s12868-019-0511-y)
Supplement: Supplementary file 11 — Additional file 11: Figure S5. Analysis of stimuli-evoked responses of GCaMP6s/+/+, GCaMP6s/orco5−/−, GCaMP6s/Gr3−/−. Maximum fluorescence values of the DE (purple) and Muscle (blue) in response to each stimulus was compared to that of water (control) to test for significance (A-C). Maximum changes in fluorescence in response to each stimulus was also compared between the DE and muscles of GCaMP6s/+/+ (green) and both GCaMP6s/orco5−/− (red) and GCaMP6s/Gr3−/− (blue) (D, E). The number of biological replicates used for each experiment were 3 or greater. *: p-value < 0.05, **: p-value < 0.01, ***: p-value < 0.001, Welch’s T-test (GCaMP6s/+/+ DE: 1-octen-3-ol n = 6; butylamine n = 11; ethyl acetate n = 10; lobeline n = 8; lactic acid n = 6; VUAA1 n = 6; sucrose n = 8; glutamate n = 5; fish food n = 4; water n = 12. GCaMP6s/+/+ Muscle: 1-octen-3-ol n = 7; butylamine n = 15; ethyl acetate n = 15; lobeline n = 13; lactic acid n = 7; VUAA1 n = 8; sucrose n = 10; glutamate n = 7; fish food n = 3; water n = 12. GCaMP6s/orco5−/− DE: 1-octen-3-ol n = 4; butylamine n = 8; ethyl acetate n = 6; lobeline n = 6; lactic acid n = 6; VUAA1 n = 4; sucrose n = 7; glutamate n = 5; fish food n = 4; water n = 7. GCaMP6s/orco5−/− Muscle: 1-octen-3-ol n = 6; butylamine n = 10; ethyl acetate n = 6; lobeline n = 6; lactic acid n = 6; VUAA1 n = 5; sucrose n = 7; glutamate n = 5; fish food n = 4; water n = 9. GCaMP6s/Gr3−/− DE: 1-octen-3-ol n = 6; butylamine n = 5; ethyl acetate n = 5; lobeline n = 4; lactic acid n = 4; VUAA1 n = 6; sucrose n = 4; glutamate n = 4; fish food n = 6; water n = 4 GCaMP6s/Gr3−/− Muscle: 1-octen-3-ol n = 7; butylamine n = 11; ethyl acetate n = 7; lobeline n = 9; lactic acid n = 6; VUAA1 n = 8; sucrose n = 7; glutamate n = 5; fish food n = 5; water n = 10). [file 12868_2019_511_MOESM11_ESM.docx]

**
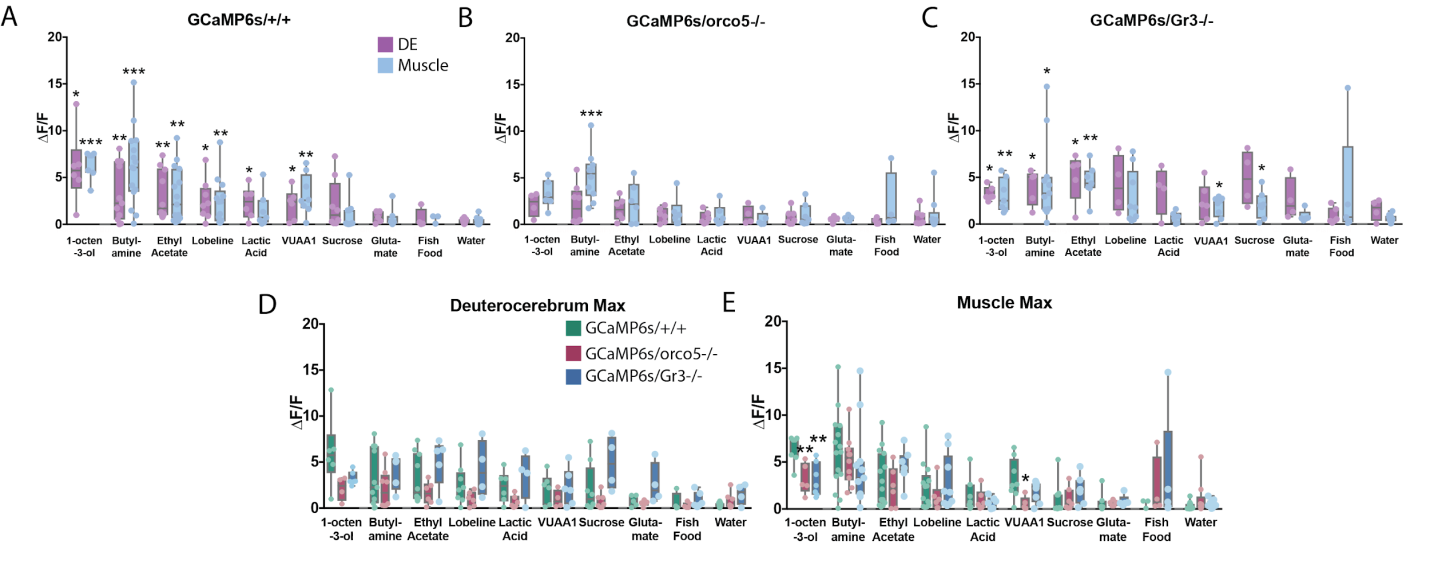
**

**Additional file 11: Figure S5. Analysis of stimuli-evoked responses of GCaMP6s/+/+, GCaMP6s/orco5-/-, GCaMP6s/Gr3-/-.** Maximum fluorescence values of the DE (purple) and Muscle (blue) in response to each stimulus was compared to that of water (control) to test for significance (A-C). Maximum changes in fluorescence in response to each stimulus was also compared between the DE and muscles of GCaMP6s/+/+ (green) and both GCaMP6s/orco5-/- (red) and GCaMP6s/Gr3-/- (blue) (D,E). The number of biological replicates used for each experiment were 3 or greater. *: p-value < 0.05, **: p-value < 0.01, ***: p-value < 0.001, Welch’s T-test (GCaMP6s/+/+ DE: 1-octen-3-ol n=6; butylamine n=11; ethyl acetate n=10; lobeline n=8; lactic acid n=6; VUAA1 n=6; sucrose n=8; glutamate n=5; fish food n=4; water n=12.

GCaMP6s/+/+ Muscle: 1-octen-3-ol n=7; butylamine n=15; ethyl acetate n=15; lobeline n=13; lactic acid n=7; VUAA1 n=8; sucrose n=10; glutamate n=7; fish food n=3; water n=12.

GCaMP6s/orco5-/- DE: 1-octen-3-ol n=4; butylamine n=8; ethyl acetate n=6; lobeline n=6; lactic acid n=6; VUAA1 n=4; sucrose n=7; glutamate n=5; fish food n=4; water n=7.

GCaMP6s/orco5-/- Muscle: 1-octen-3-ol n=6; butylamine n=10; ethyl acetate n=6; lobeline n=6; lactic acid n=6; VUAA1 n=5; sucrose n=7; glutamate n=5; fish food n=4; water n=9.

GCaMP6s/Gr3-/- DE: 1-octen-3-ol n=6; butylamine n=5; ethyl acetate n=5; lobeline n=4; lactic acid n=4; VUAA1 n=6; sucrose n=4; glutamate n=4; fish food n=6; water n=4

GCaMP6s/Gr3-/- Muscle: 1-octen-3-ol n=7; butylamine n=11; ethyl acetate n=7; lobeline n=9; lactic acid n=6; VUAA1 n=8; sucrose n=7; glutamate n=5; fish food n=5; water n=10)
