# Supplementary material for: Live calcium imaging of Aedes aegypti neuronal tissues reveals differential importance of chemosensory systems for life-history-specific foraging strategies
Source: BMC Neurosci. 2019 Jun 17;20:27. doi: 10.1186/s12868-019-0511-y (PMC6580577; doi:10.1186/s12868-019-0511-y)
Supplement: Supplementary file 12 — Additional file 12: Figure S8. Average calcium responses of GCaMP6s +/+, GCaMP/orco5−/−, and GCaMP6s/Gr3−/− over time. Responses to various stimuli were averaged over multiple replicates for each time point.(GCaMP6s/+/+ DE: 1-octen-3-ol n = 6; butylamine n = 11; ethyl acetate n = 1015; lobeline n = 813; lactic acid n = 67; VUAA1 n = 68; sucrose n = 810; glutamate n = 57; fish food n = 43; water n = 12. GCaMP6s/+/+ Muscle: 1-octen-3-ol n = 7; butylamine n = 15; ethyl acetate n = 15; lobeline n = 13; lactic acid n = 7; VUAA1 n = 8; sucrose n = 10; glutamate n = 7; fish food n = 3; water n = 12. GCaMP6s/orco5−/− DE: 1-octen-3-ol n = 46; butylamine n = 811; ethyl acetate n = 6; lobeline n = 6; lactic acid n = 6; VUAA1 n = 45; sucrose n = 7; glutamate n = 5; fish food n = 4; water n = 712. GCaMP6s/orco5−/− Muscle: 1-octen-3-ol n = 6; butylamine n = 10; ethyl acetate n = 6; lobeline n = 6; lactic acid n = 6; VUAA1 n = 5; sucrose n = 7; glutamate n = 5; fish food n = 4; water n = 9. GCaMP6s/Gr3−/− DE: 1-octen-3-ol n = 6; butylamine n = 511; ethyl acetate n = 57; lobeline n = 49; lactic acid n = 46; VUAA1 n = 68; sucrose n = 47; glutamate n = 45; fish food n = 65; water n = 412 GCaMP6s/Gr3−/− Muscle: 1-octen-3-ol n = 7; butylamine n = 11; ethyl acetate n = 7; lobeline n = 9; lactic acid n = 6; VUAA1 n = 8; sucrose n = 7; glutamate n = 5; fish food n = 5; water n = 10). [file 12868_2019_511_MOESM12_ESM.docx]

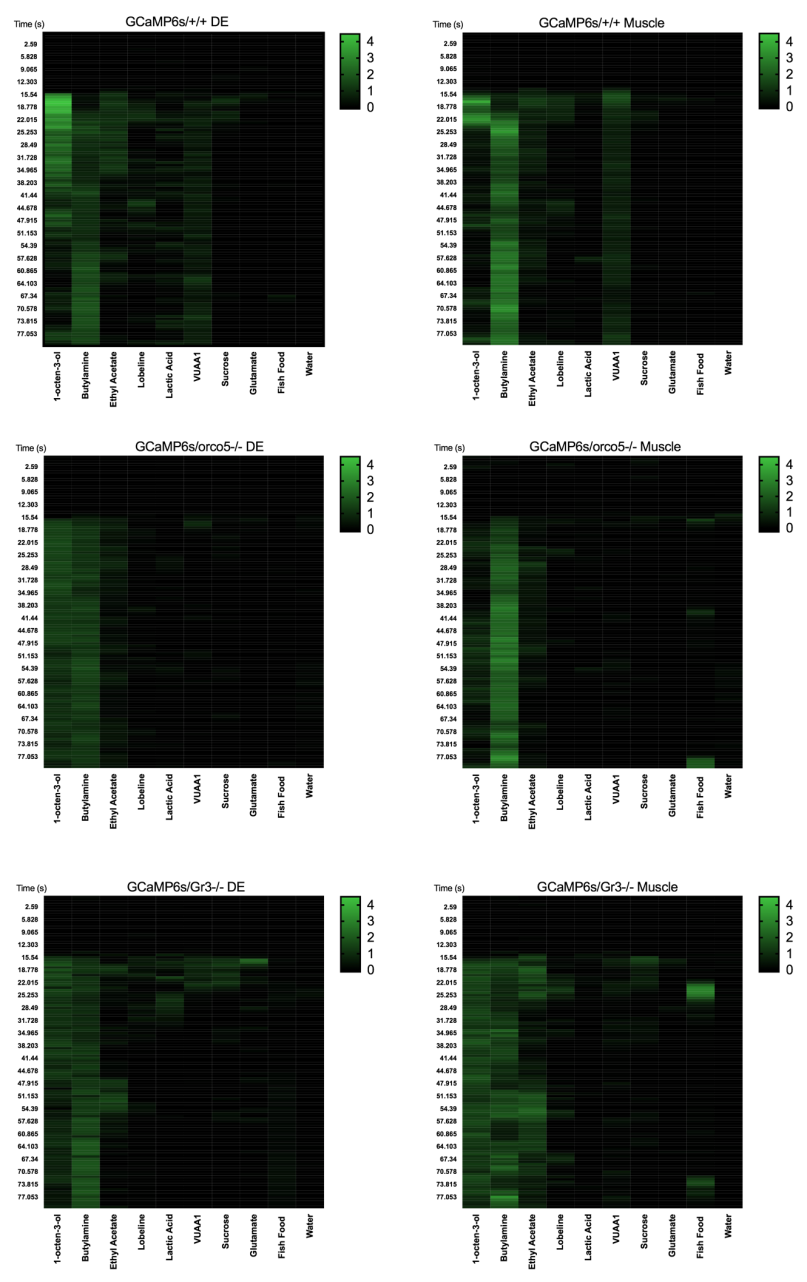


**Additional file 12: Figure S8. Average calcium responses of GCaMP6s+/+, GCaMP/orco5-/-, and GCaMP6s/Gr3-/- over time.** Responses to various stimuli were averaged over multiple replicates for each time point.(GCaMP6s/+/+ DE: 1-octen-3-ol n=6; butylamine n=11; ethyl acetate n=1015; lobeline n=813; lactic acid n=67; VUAA1 n=68; sucrose n=810; glutamate n=57; fish food n=43; water n=12.

GCaMP6s/+/+ Muscle: 1-octen-3-ol n=7; butylamine n=15; ethyl acetate n=15; lobeline n=13; lactic acid n=7; VUAA1 n=8; sucrose n=10; glutamate n=7; fish food n=3; water n=12.

GCaMP6s/orco5-/- DE: 1-octen-3-ol n=46; butylamine n=811; ethyl acetate n=6; lobeline n=6; lactic acid n=6; VUAA1 n=45; sucrose n=7; glutamate n=5; fish food n=4; water n=712.

GCaMP6s/orco5-/- Muscle: 1-octen-3-ol n=6; butylamine n=10; ethyl acetate n=6; lobeline n=6; lactic acid n=6; VUAA1 n=5; sucrose n=7; glutamate n=5; fish food n=4; water n=9.

GCaMP6s/Gr3-/- DE: 1-octen-3-ol n=6; butylamine n=511; ethyl acetate n=57; lobeline n=49; lactic acid n=46; VUAA1 n=68; sucrose n=47; glutamate n=45; fish food n=65; water n=412

GCaMP6s/Gr3-/- Muscle: 1-octen-3-ol n=7; butylamine n=11; ethyl acetate n=7; lobeline n=9; lactic acid n=6; VUAA1 n=8; sucrose n=7; glutamate n=5; fish food n=5; water n=10).
