# Supplementary material for: Live calcium imaging of Aedes aegypti neuronal tissues reveals differential importance of chemosensory systems for life-history-specific foraging strategies
Source: BMC Neurosci. 2019 Jun 17;20:27. doi: 10.1186/s12868-019-0511-y (PMC6580577; doi:10.1186/s12868-019-0511-y)
Supplement: Supplementary file 13 — Additional file 13: Figure S6. GCaMP6s/orco5−/− and GCaMP6s/Gr3−/− Line Generation and Confirmation. GCaMP6s +/+ mosquitos were mated to orco5−/− mosquitoes. Resulting individuals were mated to orco5−/− mosquitoes in a single pairwise cross for at least 8 generations and sequenced using Sanger Sequencing to confirm the presence of the orco gene (A). Mutations are indicated in red (B). GCaMP6s/+/+ mosquitos were crossed with Gr3−/− mosquitos. Individuals containing both GCaMP6s and Gr3−/− markers, dsRed/GFP transients and CFP respectively, were crossed in single pairwise matings for at least 8 generations to generate homozygous lines, individuals were then crossed to +/+ to confirm homozygosity by mendelian inheritance (C). All mosquito lines used were screened and sorted during the larval stage using a longpass-GFP and CFP filter to confirm OpIE-DsRed/GCaMP and CFP respectively (D). [file 12868_2019_511_MOESM13_ESM.docx]

**
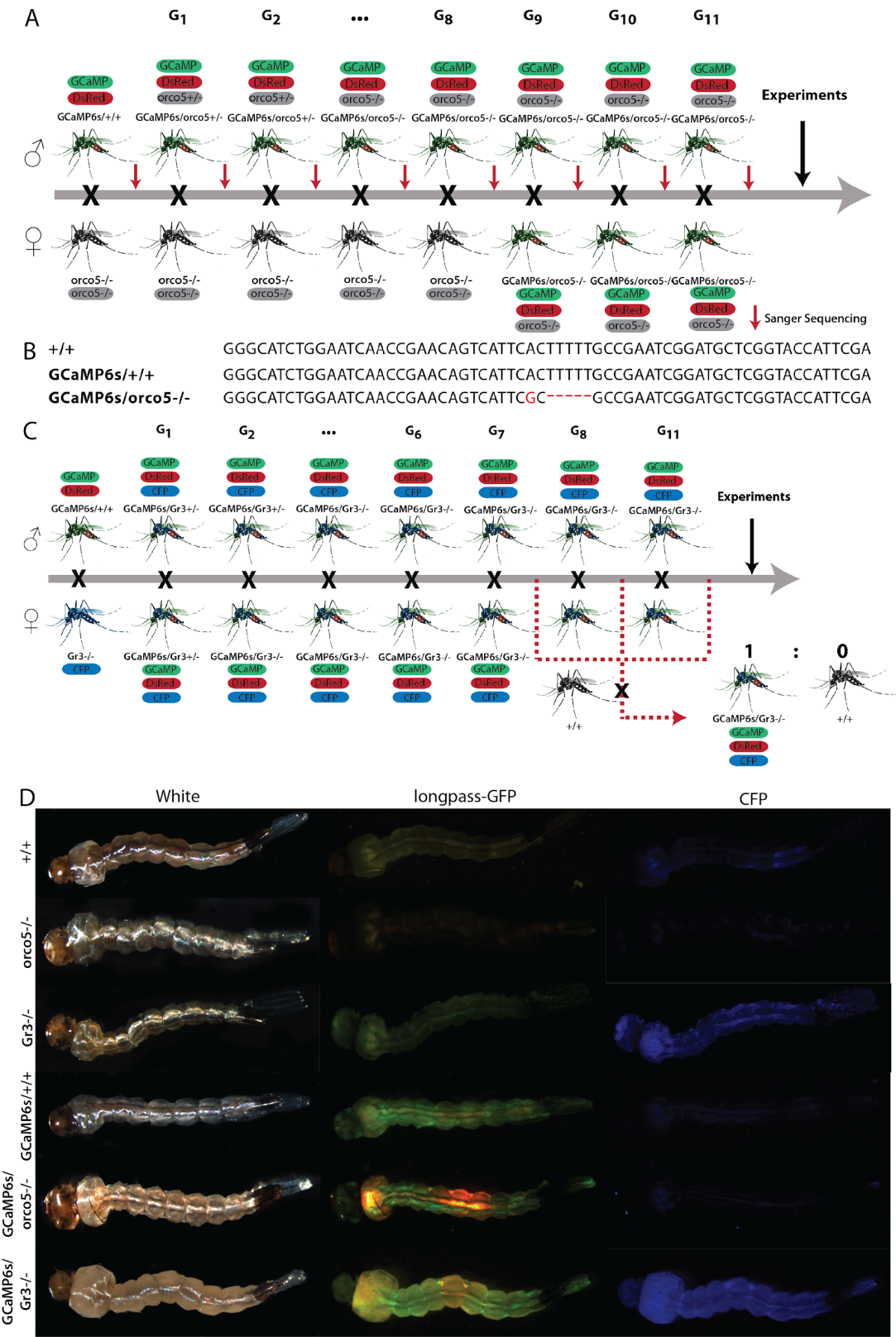
**

**Additional file 13: Figure S6. GCaMP6s/orco5-/- and GCaMP6s/Gr3-/- Line Generation and Confirmation.** GCaMP6s+/+ mosquitos were mated to orco5-/- mosquitoes. Resulting individuals were mated to orco5-/- mosquitoes in a single pairwise cross for at least 8 generations and sequenced using Sanger Sequencing to confirm the presence of the *orco* gene (A). Mutations are indicated in red (B). GCaMP6s/+/+ mosquitos were crossed with Gr3-/- mosquitos. Individuals containing both GCaMP6s and Gr3-/- markers, dsRed/GFP transients and CFP respectively, were crossed in single pairwise matings for at least 8 generations to generate homozygous lines, individuals were then crossed to +/+ to confirm homozygosity by mendelian inheritance (C). All mosquito lines used were screened and sorted during the larval stage using a longpass-GFP and CFP filter to confirm OpIE-DsRed/GCaMP and CFP respectively (D).
