# Supplementary material for: Live calcium imaging of Aedes aegypti neuronal tissues reveals differential importance of chemosensory systems for life-history-specific foraging strategies
Source: BMC Neurosci. 2019 Jun 17;20:27. doi: 10.1186/s12868-019-0511-y (PMC6580577; doi:10.1186/s12868-019-0511-y)
Supplement: Supplementary file 14 — Additional file 14: Figure S7: Larvae of different strains do not exhibit motility defects Prior to stimulation we found no differences in positional preference (A) or mean speed (B) between larvae of the mutant and wild-type backgrounds (1-way ANOVA by background, p > 0.05). Our results suggest that our arena is fair in the absence of odors (A) and that larvae of different strains do not exhibit motility defects (B). Gray bars show mean ± SEM. n = 14 ~ 24 per treatment (GCaMP6s/+/+: water n = 20, 1-octen-3-ol n = 14, food extract n = 20. GCaMP6s/orco5−/−: water n = 24, 1-octen-3-ol n = 16, food extract n = 20. GCaMP6s/Gr3−/−: water n = 16, 1-octen-3-ol n = 17, food extract n = 16, Liverpool wt: water n = 19). [file 12868_2019_511_MOESM14_ESM.docx]

**
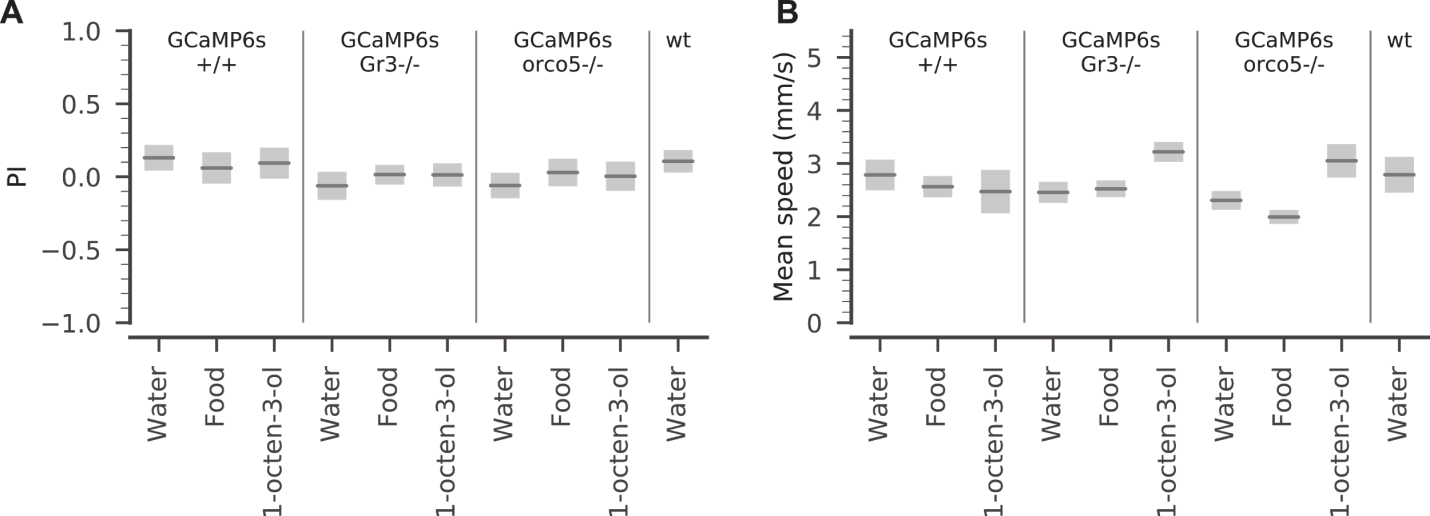
**

**Additional file 14: Figure S7: Larvae of different strains do not exhibit motility defects**

Prior to stimulation we found no differences in positional preference (A) or mean speed (B) between larvae of the mutant and wild-type backgrounds (1-way ANOVA by background, p>0.05). Our results suggest that our arena is fair in the absence of odors (A) and that larvae of different strains do not exhibit motility defects (B). Gray bars show mean +/- SEM. n = 14 ~ 24 per treatment (GCaMP6s/+/+: water n=20, 1-octen-3-ol n=14, food extract n=20. GCaMP6s/orco5-/-: water n=24, 1-octen-3-ol n=16, food extract n=20. GCaMP6s/Gr3-/-: water n=16, 1-octen-3-ol n=17, food extract n=16, Liverpool wt: water n=19).
