# Supplementary material for: Live calcium imaging of Aedes aegypti neuronal tissues reveals differential importance of chemosensory systems for life-history-specific foraging strategies
Source: BMC Neurosci. 2019 Jun 17;20:27. doi: 10.1186/s12868-019-0511-y (PMC6580577; doi:10.1186/s12868-019-0511-y)
Supplement: Supplementary file 15 — Additional file 15: Table S2. Primer Sequences used in this study. [file 12868_2019_511_MOESM15_ESM.docx]

**Additional file 15: Table S2. Primer Sequences Used in this study**

| Primer name | Primer sequence (5’ to 3’) | Source |
| --- | --- | --- |
| 997.C1  997.C2 | CGACGGTCACGGCGGGCATGTCGACGCGGCCGCTATCTTTACATGTAGCTTGTGCATTGA  AGCCATACCATGATGATGATGATGATGAGAACCCATCTCGAGATTCGTTGAAATCTCTGT | *Ae. aegypti* genomic DNA |
| 997.C3  997.C4 | TTTTCTGCTCAACAGAGATTTCAACGAATCTCGAGATGGGTTCTCATCATCATCATCATC  GTCAGATCCGAGATCGGCCGGCCTAGGGCGCGCCTTAATTAATCACTTCGCTGTCATCAT | Addgene plasmid #740753 |
| 997.C5  997.C6 | CGGTATCTCGCGTTTGTTTGATCGCACGGTTCCCACAATGGTTAATTCGAGCTCGCCCGG  ATTGGATTCAATGCACAAGCTACATGTAAAGATAGCGGCCGCGTCGACATGCCCGCCGTG | attB plasmid |
| 997.C7  997.C8 | GTTTGTACAAATGATGACAGCGAAGTGATTAATTAACTAGAATGAATCGTTTTTAAAATA  AAAAAGTTGGTGGTGGGGAGGCCACCGAGTATGGGCGCGCCCCGGCCGTTAACTCGAATC | Addgene plasmid 36432 |
| 997.C9  997.C10 | TGGCTTGGATAGCGATTCGAGTTAACGGCCGGGGCGCGCCCATACTCGGTGGCCTCCCCA  GCATGAACTCCTTGATGACGTTCTTGGAGGAGCGCACCATCACCAGAGACAGGTTGCGGC | Invitrogen pIZ/V5-His/CAT |
| 997. C11  997. C12 | GCTAACGGCAAACACCATAAC  CGAAGAAAGCTCTCAGGTAACA | orco5-/- confirmation primers |
