# Supplementary material for: Live calcium imaging of Aedes aegypti neuronal tissues reveals differential importance of chemosensory systems for life-history-specific foraging strategies
Source: BMC Neurosci. 2019 Jun 17;20:27. doi: 10.1186/s12868-019-0511-y (PMC6580577; doi:10.1186/s12868-019-0511-y)
Supplement: Supplementary file 16 — Additional file 16: Table S3. Inverse PCR Primer sequences used in this study. [file 12868_2019_511_MOESM16_ESM.docx]

**Additional file 16: Table S3. Inverse PCR Primer sequences used in this study**

| **Reaction** | **Primer Name** | **Primer Sequence (5’ - 3’)** |
| --- | --- | --- |
| 5’ (1st Round PCR) | 991.5F1 | GACGCATGATTATCTTTTACGTGAC |
|  | 991.5R1 | TGACACTTACCGCATTGACA |
| 5’ (2nd Round PCR) | 991.5F2 | GCGATGACGAGCTTGTTGGTG |
|  | 991.5R2 | TCCAAGCGGCGACTGAGATG |
| 3’ (1st Round PCR) | 991.3F1 | CAACATGACTGTTTTTAAAGTACAAA |
|  | 991.3R1 | GTCAGAAACAACTTTGGCACATATC |
| 3’ (2nd Round PCR) | 991.3F2 | CCTCGATATACAGACCGATAAAAC |
|  | 991.3R2 | TGCATTTGCCTTTCGCCTTAT |
| Confirmation of 3’ | 1018.S6 | CAGGCGCTGGAAAAATAATGTGAG |
|  | 1018.S7 | CTCACATTATTTTTCCAGCGCCTG |
| Confirmation of 5’ | 1018.S8 | TTTCCACGAAATGAACTCAAACGC |
|  | 1018.S9 | CACACTAATGTACAGTCAGTCTATGCTACGC |
|  | 1018.S10 | AGAAGAACAGAGGCAATCAACTACATTGA |
